# An immunity and pyroptosis gene-pair signature predicts overall survival in acute myeloid leukemia

**DOI:** 10.1038/s41375-022-01662-6

**Published:** 2022-08-09

**Authors:** Weikaixin Kong, Liye He, Jie Zhu, Oscar Brück, Kimmo Porkka, Caroline A. Heckman, Sujie Zhu, Tero Aittokallio

**Affiliations:** 1 Institute for Molecular Medicine Finland (FIMM), HiLIFE, University of Helsinki, Helsinki, Finland; 2 Peking University Health Science Center, Department of Pharmacology, School of Basic Medical Sciences, Peking University, Beijing, China; 3 Institute of Translational Medicine, The Affiliated Hospital of Qingdao University, College of Medicine, Qingdao University, Qingdao, China; 4 Helsinki University Hospital Comprehensive Cancer Center, Department of Hematology, Helsinki, Finland; 5 iCAN Digital Precision Cancer Medicine Flagship, University of Helsinki, Helsinki, Finland; 6 Institute for Cancer Research, Department of Cancer Genetics, Oslo University Hospital, Oslo, Norway; 7 Oslo Centre for Biostatistics and Epidemiology (OCBE), Faculty of Medicine, University of Oslo, Oslo, Norway

**Keywords:** Risk factors, Acute myeloid leukaemia

## Abstract

Treatment responses of patients with acute myeloid leukemia (AML) are known to be heterogeneous, posing challenges for risk scoring and treatment stratification. In this retrospective multi-cohort study, we investigated whether combining pyroptosis- and immune-related genes improves prognostic classification of AML patients. Using a robust gene pairing approach, which effectively eliminates batch effects across heterogeneous patient cohorts and transcriptomic data, we developed an immunity and pyroptosis-related prognostic (IPRP) signature that consists of 15 genes. Using 5 AML cohorts (*n* = 1327 patients total), we demonstrate that the IPRP score leads to more consistent and accurate survival prediction performance, compared with 10 existing signatures, and that IPRP scoring is widely applicable to various patient cohorts, treatment procedures and transcriptomic technologies. Compared to current standards for AML patient stratification, such as age or ELN2017 risk classification, we demonstrate an added prognostic value of the IPRP risk score for providing improved prediction of AML patients. Our web-tool implementation of the IPRP score and a simple 4-factor nomogram enables practical and robust risk scoring for AML patients. Even though developed for AML patients, our pan-cancer analyses demonstrate a wider application of the IPRP signature for prognostic prediction and analysis of tumor-immune interplay also in multiple solid tumors.

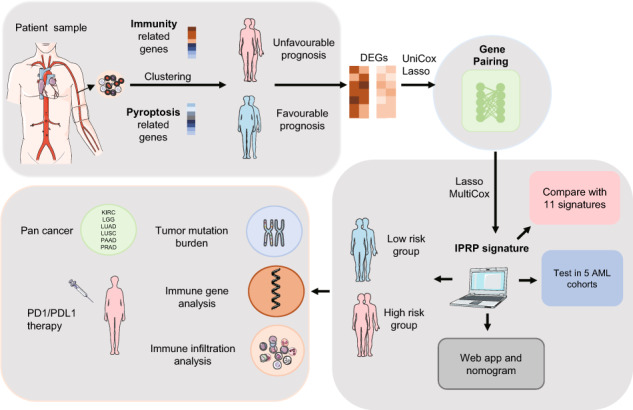

## Introduction

Pyroptosis is a process of programmed death of inflammatory cells, mainly operating through the activation of a variety of caspases mediated by inflammasomes. Pyroptosis causes shearing and multimerization of various gasdermin family members, including gasdermin D (GSDMD), resulting in cell perforation, which in turn leads to cell death [1]. Compared with apoptosis, cell pyroptosis occurs faster and it is accompanied by the release of a large number of pro-inflammatory factors [2]. Therefore, cell pyroptosis is closely related to the immune process, but it is also essential for tumor development. Recent studies have shown that pyroptosis-related genes are associated with the prognosis of cancer patients. For example, Ye et al. [3] found that a pyroptosis-related signature can effectively predict the prognosis of patients with ovarian cancer. Shao et al. [4] proposed a pyroptosis signature, which is closely related to the degree of immune infiltration in gastric cancer patients. Similarly, Ju [5] and Lin [6] et al. found that pyroptosis-related genes can be used for prognostic prediction of skin cutaneous melanoma and lung adenocarcinoma, respectively. These studies indicate the importance of the pyroptosis process in tumor development and treatment responses. However, whether pyroptosis also plays a role in hematological cancers remains an open question. Compared with solid tumor sampling, the bone marrow or peripheral blood of leukemia patients is easier to access, and therefore an accurate and practical prognostic signature for leukemia patients has potential for more direct clinical application. Since most hematological malignancies are very heterogeneous diseases, robust patient-specific prognostic signatures are required for clinical applications.

Inconsistent data formats and batch effects between different profiling platforms often challenge the development and application of prognostic gene signatures. For example, transcriptomic data from microarrays and RNA sequencing have rather distinct distributional properties. Data sets profiled in various patient cohorts may also have been processed with different data processing methods, making the signatures extracted in one study suboptimal in the other cohorts. In particular, using traditional prognostic signatures, it is often difficult to find a common cutoff for risk classification that would be suitable among multiple patient cohorts and studies. To make the gene signature and risk cutoff widely applicable, we developed a novel analytic approach based on mRNA expression levels of gene-pairs to construct a robust prognostic model, and show how one-hot binary encoding of the gene-pairs effectively eliminates the influence of batch effects. To demonstrate the robustness of the gene pairing approach, we apply it here for the first time to establish a gene-paired immunity and pyroptosis related prognostic (IPRP) signature for AML patients. In comparison with ten existing signatures, we show how the IPRP signatures provides a universally applicable risk score for heterogeneous AML datasets and cohorts of patients, profiled with different transcriptomic platforms. Even if developed in AML patient cohorts, our pan-cancer analyses show its wider application for survival prediction and analysis of tumor immunity in multiple cancer types.

## Materials/Subjects and Methods

### AML patient cohorts and gene expression data

To develop a robust prognostic signature for AML patients, we used 5 AML cohorts, that contained a total of 1327 patients, and associated gene expression data sets (Supplementary Table 1, Supplementary Fig. 1, and Supplementary Data).

### Differential expression analysis

To integrate both pyroptosis and immune processes in the signature development, we identified differentially expressed genes (DEGs) between the double favorable prognosis group (FF) and double unfavorable prognosis (UU) groups. The screening criteria for DEGs were |log_2_FC | >1 and FDR < 0.001 (Wilcoxon test). Finally, we obtained 3720 DEGs, and these genes were used for subsequent feature selection.

### Feature selection and model building

Among the 3720 DEGs, univariate Cox regression and LASSO regularized regression were used to reduce the number of features to 26 genes (see Supplementary Fig. 2). To construct the paired IPRP signature, we paired these 26 genes after the first LASSO regression and applied one-hot binary encoding: if expression of gene *A* > expression of gene *B*, then the feature “Gene A | Gene B” was marked as 1, and otherwise it is marked as 0, as shown in Eq. (1).

1 $${{{{{{{\mathrm{Gene}}}}}}}}\;{{{{{{{\mathrm{A|Gene}}}}}}}}\;{{{{{{{\mathrm{B}}}}}}}} = \left\{ {\begin{array}{*{20}{c}} {1,Expression\left( A \right) \, > \,Expression\left( B \right)} \\ {0,Expression\left( A \right) \le Expression\left( B \right)} \end{array}} \right.$$

We deleted the gene-pairs with frequency of the “1” label in the training set less than 0.2 or greater than 0.8, since such features do not contain significant classification information. Using this paired approach, we obtained 112 gene-pairs as features, which were further reduced by the second LASSO regression. Finally, multivariate Cox regression (with “both” option in the feature selection) was used to construct the IPRP signature which contains 10 gene-pairs. The above analyses were carried out using “glmnet”, “survival” and “survminer” packages in R.

### Validation of the prognostic model

To demonstrate the robustness and added values of the paired IPRP signature of 10 gene-pairs, we compared its performance against the unpaired IPRP signature of 26 genes, and against 10 other signatures that were used as comparison models, namely Pyroptosis signature, IRG signature [7], Autophagy signature [8], Hypoxia signature [9], CXCR signature [10] and 24-gene signature [11], LSC17 signature [12], 7-gene signature [13], pLSC6 signature [14], and PS29MRC signature [15]. We used the following criteria for selection of these signatures: (i) The signature was constructed based on key biological processes of AML or using a meta-analysis of existing signatures. (ii) The genes in the signature had most of the expression levels measured in the 5 AML datasets.

### Gene mutation and immune profile analyses in AML cohorts

Since the TCGA-AML and BeatAML datasets include comprehensive gene mutation data, the analysis of point mutations and tumor mutation burden (TMB) was carried out in these two data sets. The point mutation data were obtained from the GDC Data Portal (https://portal.gdc.cancer.gov/) and BeatAML study [16], respectively. The samples with mutation data were intersected with the samples with the expression data (Supplementary Fig. 1), which led to 104 patient samples in TCGA-AML and 261 patient samples in BeatAML containing both genome-wide expression data and point mutation data. For these patients, we used the “maftools” package in R to make a waterfall plot (that only displays top-20 mutations with the highest frequency), and calculated the TMB by the number of non-synonymous somatic mutations (single nucleotide variants and small insertions/deletions) per million bases in the coding region. Please see Supplementary Data for more details.

### Pan-cancer analysis of overall survival and tumor immunity

To explore the wider value of IPRP score in other types of cancers, data from 33 different cancer types in TCGA were used for pan-cancer analysis. The TCGA pan-cancer data were downloaded from the UCSC Xena database (https://xena.ucsc.edu/), out of which 10,071 cancer patients have complete expression profiles, point mutation data, overall survival (OS) and survival status. These data were used to calculate the IPRP score and to perform Cox prognostic analysis Benjamini-Hochberg correction to calculate the hazard ratio. TMB has been shown to be associated with the efficacy of immunotherapy in multiple caner types [17, 18]. Since AML is a cancer with low TMB, it is expected to have a low reference value for immunotherapy in AML. Instead, we explored the interplay between immunotherapy, TMB and IPRP score in multiple solid tumors using the TCGA data, where we conducted a pan-analysis of the expression levels of immunotherapy-related targets *CD47*, *CTLA4* and *CD274* (*PD-L1*).

## Results

### Clustering of AML patients based on pyroptosis and immune-related genes

**Fig. 1:**
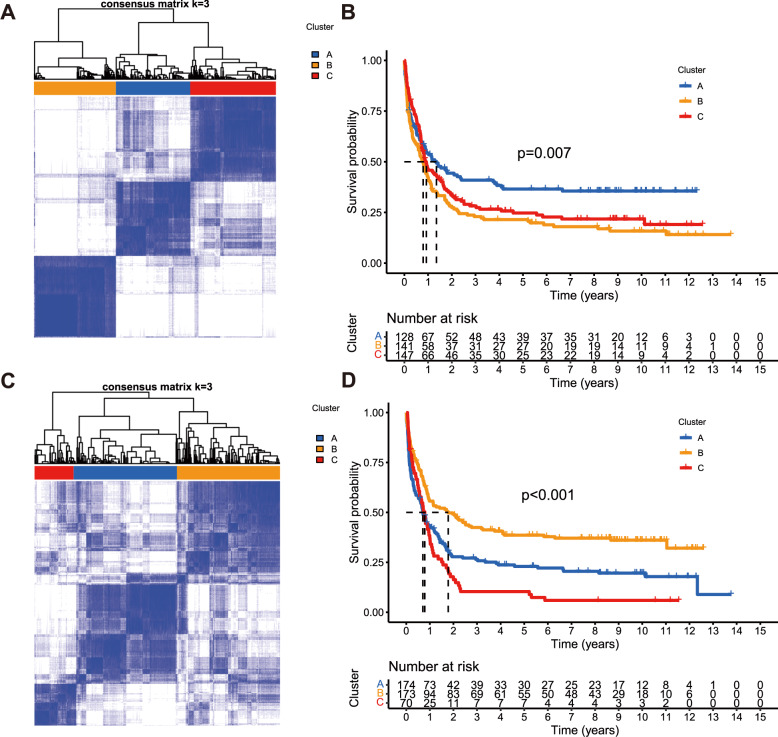
Clustering of the AML patients in the training dataset (*n* = 417) based on their gene expression profiles. **A** Clustering heatmap based on 22 pyroptosis related genes in the AML training cohort. **B** Survival curves of patients in the pyroptosis-related clusters. Survival differences were assessed with Log Rank test (cluster A compared with clusters B and C). Censored data at the last follow-up point was excluded when plotting the survival curve of cluster A. **C** Clustering heat map based on the 156 immune-related genes in the AML training cohort. **D** Survival curves of patients in the immune-related clusters. Survival differences were assessed with Log Rank test (cluster B compared with clusters A and C).

To construct and validate the IPRP signature, we used 5 AML patient cohorts with genome-wide transcriptomic profiles (Supplementary Figs. 1, 2 and Supplementary Table 1). We first clustered the AML patients in the training dataset using the 22 pyroptosis and 156 immunity-related genes that distinguished the survival status of the AML patients in the training data (Fig. 1; *p* < 0.05; Log Rank test). There were no overlapping genes between the immune and pyroptosis-related genes, which ensure the orthogonality of the two clustering solutions. In both clustering analyses, the increased area under the consensus CDF curves was used for choosing the number (k) of clusters (Supplementary Fig. 3), and k = 3 was found to provide the optimal number of clusters based on the relative increase in the area under the consensus CDF curves (Supplementary Fig. 3B, D).

The clustering results based on the pyroptosis-related genes show that the patients in cluster A have more favorable prognosis, when compared to the patients in clusters B and C (Fig. 1A, B; *p* = 0.007, Log Rank test). Similarly, the clustering results based on the immunity-related genes show that the patients in cluster B have more favorable prognosis, compared to the patients in clusters A and C (Fig. 1C, D; *p* < 0.001, Log Rank test). Even though the two gene sets were distinct, there was significant consistency in the patient prognostic classification based on the pyroptosis and immunity processes (Supplementary Table 2; *p* < 0.001, Chi-square test), motivating their integration for improved performance; in particular, there were 73 patients with favorable prognosis based on both gene sets (FF group), and 192 patients with unfavorable prognosis based on both gene sets (UU group, Supplementary Table 2).

We next studied the differential expression between the prognostic groups and obtained 3720 DEGs between the FF and UU groups (FDR < 0.001, Wilcoxon test), of which 2140 genes were up-regulated and 1580 genes were down-regulated in the FF group (log |FC| > 1). To further explore the biological function of these genes, we performed GSVA analysis of differentially activated pathways. In the GSVA results, 19 of the top-20 KEGG pathways were significantly activated in the FF group (FDR < 0.05, Wilcoxon test), whereas only the ABC transporter pathway was down-regulated in the FF group (Supplementary Fig. 4). Since high expression of ABC transporter is associated with drug resistance [19], the inhibition of this pathway in the FF group partly explains the better prognosis of the AML patients in that group. In addition, pathways related to glycolysis were also significantly activated in the FF group, including glycosaminoglycan degradation and glycolysis gluconeogenesis.

Interestingly, the activated pathways in the FF group were implicated in 7 different cancers, with various tissue origins, both hematological and solid tumors, yet still closely clustered with the AML pathway activity (Supplementary Fig. 4). This indicates that these cancers share similar gene expression programs that govern the pyroptosis and immune-related processes.

### Construction of the IPRP signature for prognostic classification of AML patients

**Fig. 2:**
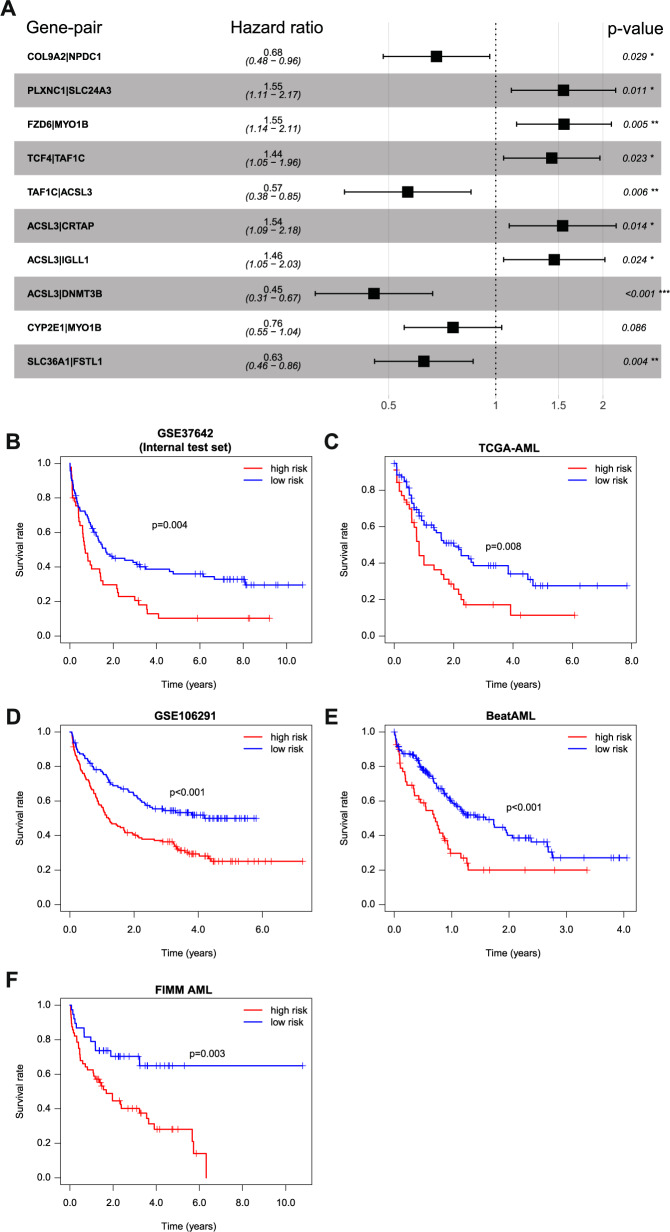
Construction and validation of the paired IPRP signature and risk score in AML patients. **A** The hazard ratios of the IPRP signature genes based on multivariate Cox regression in the training set. *p < 0.05, **p < 0.01, ***p < 0.001 (Wald’s test). Survival curves in the IPRP high and low-risk groups in **B** GSE37642 (internal test set), **C** TCGA-AML, **D** GSE106291, **E** BeatAML, **F** FIMM AML cohorts. The median value of the IPRP risk score in the training set (0.684) was used as the cutoff to separate between the high risk and the low risk patient groups in all the AML cohorts. Statistical significance of the survival differences between the two groups was assessed with Log Rank test.

Using univariate Cox regression, we first selected 763 genes out of the 3720 DEGs between the FF and UU groups, which were associated with survival differences in the training set (Supplementary Fig. 2; *p* < 0.05, Wald’s test and Benjamini-Hochberg multiple testing correction). In the next step, we used multivariate penalized LASSO regression to further select 26 genes most predictive of the survival status using optimal penalty coefficient (Supplementary Fig. 5A, B). These 26 genes were subjected to gene-pairing, resulting in a total of 325 (25 × 26/2) gene pairs; 112 of these pairs had a frequency of “gene *A* > gene *B* expression” between 20% and 80% in the training set, which were considered to have sufficient information content. In the gene-pair LASSO regression (Supplementary Fig. 2), 30 of the 112 gene-pairs were selected for survival prediction in the training set (Supplementary Fig. 5C, D). Finally, using multivariate Cox regression, there remain 10 gene-pairs (among 15 genes) associated with survival differences, which formed the IPRP signature (Fig. 2A, Supplementary Table 3); these 10 gene pairs include 5 risk factors (HR > 1) and 5 protective factors (HR < 1). Therefore, the calculation of the IRPR risk score is based on:

$${{{{{{{\mathrm{Sum}}}}}}}} =	\, {- 0.384} \times {{{{{{{\mathrm{COL}}}}}}}}9{{{{{{{\mathrm{A}}}}}}}}2\left| {{{{{{{{\mathrm{NPDC}}}}}}}}1 + 0.438 \times {{{{{{{\mathrm{PLXNC}}}}}}}}1} \right|{{{{{{{\mathrm{SLC}}}}}}}}24{{{{{{{\mathrm{A}}}}}}}}3 + 0.441\\ 	\times {{{{{{{\mathrm{FZD}}}}}}}}6\left| {{{{{{{{\mathrm{MYO}}}}}}}}1{{{{{{{\mathrm{B}}}}}}}} + 0.362 \times {{{{{{{\mathrm{TCF}}}}}}}}4} \right|{{{{{{{\mathrm{TAF}}}}}}}}1{{{{{{{\mathrm{C}}}}}}}} - 0.569\\ 	 \times {{{{{{{\mathrm{TAF}}}}}}}}1{{{{{{{\mathrm{C}}}}}}}}\left| {{{{{{{{\mathrm{ACSL}}}}}}}}3 + 0.433 \times {{{{{{{\mathrm{ACSL}}}}}}}}3} \right|{{{{{{{\mathrm{CRTAP}}}}}}}} + 0.378\\ 	 \times {{{{{{{\mathrm{ACSL}}}}}}}}3\left| {{{{{{{{\mathrm{IGLL}}}}}}}}1 - 0.788 \times {{{{{{{\mathrm{ACSL}}}}}}}}3} \right|{{{{{{{\mathrm{DNMT}}}}}}}}3{{{{{{{\mathrm{B}}}}}}}} - 0.277 \\ 	 \times {{{{{{{\mathrm{CYP}}}}}}}}2{{{{{{{\mathrm{E}}}}}}}}1|{{{{{{{\mathrm{MYO}}}}}}}}1{{{{{{{\mathrm{B}}}}}}}} - 0.465 \times {{{{{{{\mathrm{SLC}}}}}}}}36{{{{{{{\mathrm{A}}}}}}}}1|{{{{{{{\mathrm{FSTL}}}}}}}}1$$

$${{{{{{{\mathrm{RiskScore}}}}}}}} = e^{Sum}$$

To investigate the prognostic performance of the 10 gene-pair IPRP signature, we applied the risk score estimated in the training data to 5 independent AML patient cohorts (4 external cohorts and 1 internal test cohort, see Supplementary Fig. 1), which were not used in the construction of the IPRP signature. To mimic a future application of the signature into new patient cohorts, we used the median value of IPRP risk score in the training set (0.684) as the cutoff to separate between the high risk and the low risk patients also in the other cohorts. Notably, the unified IPRP risk score showed a wide prognostic value in distinguishing the survival status of the AML patients across the AML patient cohorts (Fig. 2B–G, Supplementary Fig. 6). This is a rather striking result, given the wide heterogeneity of the disease and of the AML cohorts in terms of differences in patient characteristics, treatment procedures, follow-up times and transcriptomic platforms (Supplementary Table 1). Consistently, the high risk group had a worse prognosis in all the cohorts, indicating that the risk score based on the paired IPRP signature of only 10 gene-pairings is a robust prognostic factor in AML.

### Comparative evaluation of the IPRP signature against other signatures

**Fig. 3:**
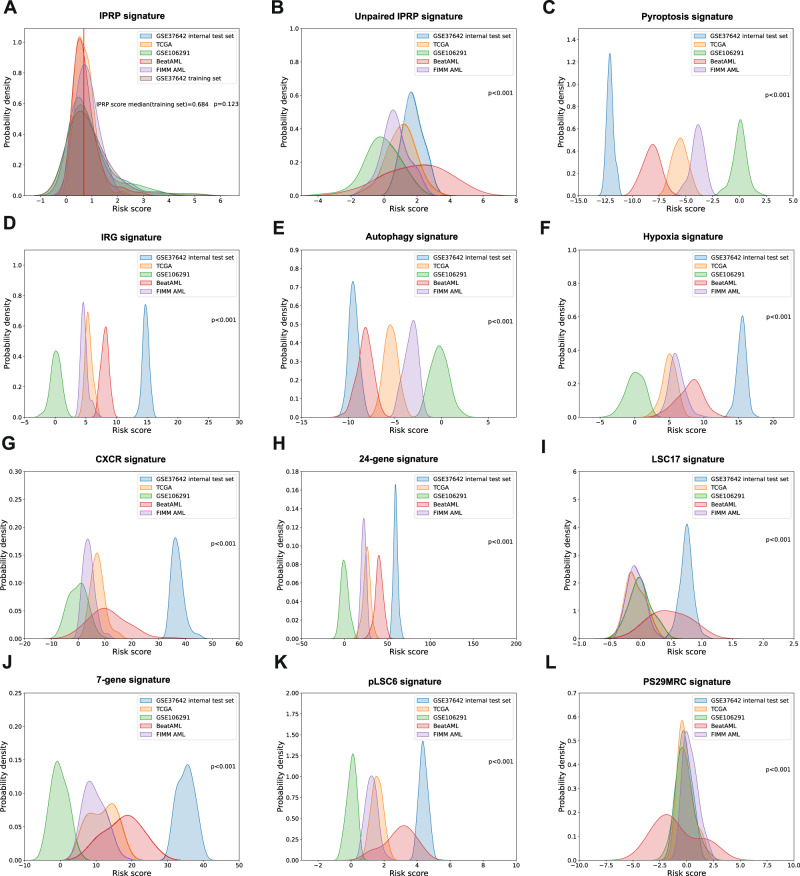
The distributions of risk scores calculated based on various prognostic signatures across the AML cohorts. **A** Paired IPRP signature. **B** Unpaired IPRP signature, **C** Pyroptosis signature, **D** IRG signature, **E** Autophagy signatures, **F** Hypoxia signature, **G** CXCR signature, **H** 24-gene signature, **I** LSC17 signature, **J** 7-gene signature, **K** pLSC6 signature, **L** PS29MRC signature. The differences between the score distributions across the cohorts were assessed with ANOVA test (not a formal statistical hypothesis testing).

To further study the sources of robustness and added value of the paired IPRP signature, we compared its performance against an unpaired IPRP signature, pyroptosis-only signature and 10 existing prognostic signatures for AML (see Methods). To study the robustness of the signatures, we calculated the risk scores based on these signatures in the internal test set and 4 external AML patient cohorts (Fig. 3). Compared with the distribution of the IPRP risk score (Fig. 3A), the other risk scores were distributed relatively differently across the AML cohorts (*p* < 0.001, ANOVA test), due to lack of robustness to the various characteristics of the datasets and cohorts, which makes it difficult to determine a common risk cutoff applicable in various data sets and cohorts. In contrast, the risk score calculated based on the paired IPRP signature resulted in similar distributions, regardless of the AML cohort or whether RNA-seq or microarray platform was used for transcriptomic profiling (*p* = 0.123, ANOVA test). This demonstrates that the signature and risk score from the gene-pairing approach is less sensitive to differences in the characteristics of data cohort. See Supplementary Data for more results of the effects of batch effects.

**Fig. 4:**
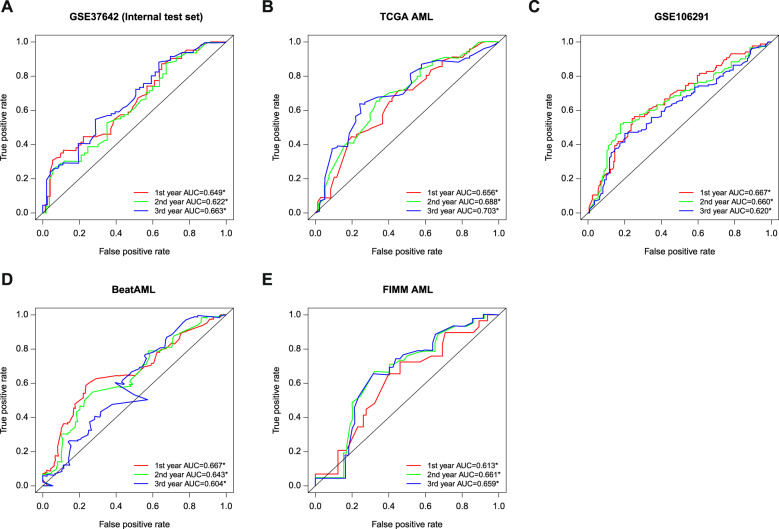
The prognostic performance of IPRP score across AML test cohorts. The receiver operating characteristic (ROC) curves of the paired IPRP score in **A** GSE37642 (internal test set), **B** TCGA-AML, **C** GSE106291, **D** BeatAML, **E** FIMM AML cohorts. The colored curves correspond to different follow-up time points in the survival data with ROC-AUC values. The black diagonal line corresponds to the random classifier (ROC-AUC = 0.5). *p < 0.05, permutation test.

**Fig. 5:**
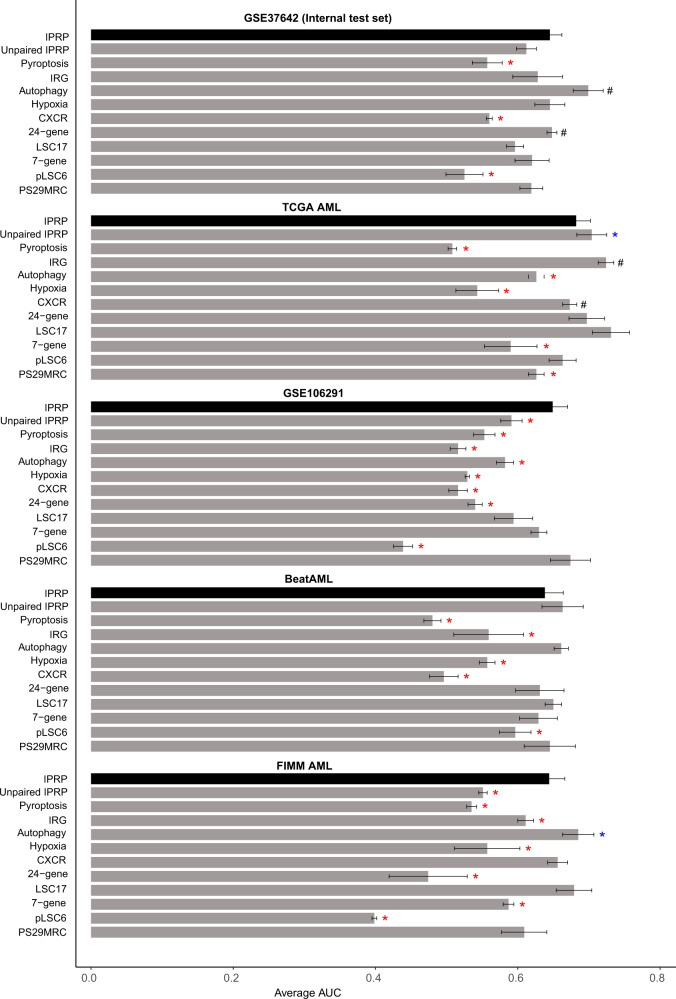
Comparison of the paired IPRP risk score and the other risk scores in AML patient cohorts. The bars indicate average ROC-AUC and SD over the 1, 2, and 3-year time points. The statistical significance was calculated using paired Wilcoxon test. Red and blue * indicate that the signature had either a lower or higher average ROC-AUC (*p* < 0.05, Wilcoxon test), compared to IPRP risk score, respectively. ^#^ Indicates that the signature was originally developed in that particular patient cohort.

We further compared the paired IPRP model against the existing 10 signatures in terms of their survival prediction accuracy using AUC-ROC analyses and permutation test (Figs. 4, 5, and Supplementary Table 6). The paired IPRP signature achieved its best AUC performance in the external TCGA-AML dataset (Fig. 4). When averaging over the follow-up time points, the IPRP signature showed consistently accurate prognostic classification results, compared to many of the existing signatures (Fig. 5). Even though some of the other signatures resulted in a relatively high prognostic accuracy in a particular cohort, especially in those they were established, they did not lead to comparable accuracy in other cohorts. For instance, the Autophagy signature had a good performance in GSE37642, where it was trained, and in the FIMM AML cohort, but it showed significantly poorer performance in TCGA-AML and GSEA 106219 datasets. These results show that the paired IPRP signature provides a robust prognostic risk score for AML patients (Supplementary Table 6).

### Establishment of a web-based nomogram for AML risk scoring

**Fig. 6:**
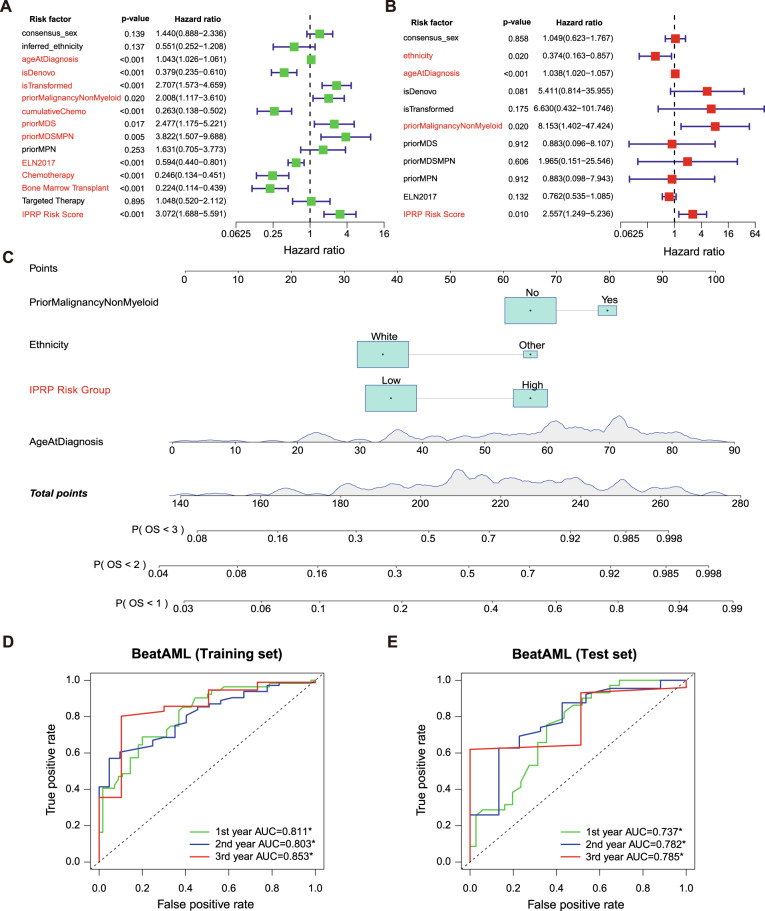
The establishment and validation of risk nomogram in the BeatAML data set. **A** Univariate independent prognostic analysis. **B** Multivariate independent prognostic analysis based on variables at the diagnosis stage. **C** 4-factor nomogram containing the paired IPRP risk score. **D** The prognostic prediction accuracy in the BeatAML training set (*n* = 152). **E** The prognostic prediction accuracy in the BeatAML test set (*n* = 72). The colored curves correspond to different follow-up time points in the survival data with ROC-AUC values. The black diagonal line corresponds to the random classifier (ROC-AUC = 0.5). *p < 0.05, permutation test.

Toward more routine clinical application, we built an easy-to-use nomogram based on the paired IPRP score for AML risk scoring using the data from the BeatAML cohort. To investigate whether the paired IPRP score is an independent risk factor, we first divided the BeatAML samples with complete clinical information into a training set (*n* = 152) and a test set (*n* = 72). Univariate independent prognostic analysis in the training set showed that age at diagnosis along with several other clinical factors and IPRP risk score were independent prognostic factors for AML patient survival (Fig. 6A; *p* < 0.05, Wald’s test); however, in multivariate prognostic analysis, when considering the variables available at diagnosis, only the age at diagnosis, prior non-myeloid malignancy, ethnicity, and IPRP risk score remained as independent risk factors (Fig. 6B; *p* < 0.05, Wald’s test). The cumulative sum of points from the remaining four factors, estimated in the BeatAML training set, can be used to infer the risk of 1-, 2-, and 3-year mortality of a patient based on the nomogram (Fig. 6C).

To verify the performance of the risk scoring nomogram, we first carried out risk classification analyses in the BeatAML cohort, where the 4-factor nomogram achieved the AUC-ROC values for 1-, 2-, and 3-years survival as 0.811, 0.803, and 0.853 in training set, and 0.737, 0.782, and 0.785 in BeatAML test set, respectively (*p* < 0.05, permutation test; Fig. 6D, E). Notably, the accuracy of the 4-factor nomogram was improved compared to that when using the IPRP score alone (Supplementary Fig. 11A, B). Similar improvements were observed also in the TCGA-AML cohort (Supplementary Fig. 11C, D), and in the FIMM AML cohort (Supplementary Fig. 11E, F). These results show that the 4-factor nomogram, which includes the paired IPRP score, enables relatively accurate and simple prognostic prediction of AML patients. We note that the prediction accuracies for individual patients are not always perfect, as can be expected in such a challenging prediction problem.

As expected, the age at diagnosis played a key role in the nomogram, with a wide point range (Fig. 6C), and it had significant association with the IPRP score (*p* < 0.001), even though the correlation coefficient was modest (R = 0.24; Supplementary Fig. 12B). This indicates that the IPRP score provides additional prognostic information compared to age and other patient characteristics. Interestingly, ELN2017 risk classification, that is clinically used as a prognostic tool for AML patients, was not identified as independent risk factor in the multivariate prognostic analysis (*p* = 0.132; Fig. 6B). Including ELN2017 as an additional factor into the nomogram and reconstructing the nomogram (Supplementary Fig. 12A) did not improve its prediction accuracy obviously either in the BeatAML or FIMM AML cohorts (Supplementary Fig. 13), likely due to the significant and expected association between IPRP score and ELN2017 classification (*p* < 0.001, ANOVA test; Supplementary Fig. 12C).

To make the nomogram widely applicable, we implemented it as a stand-alone and easy-to-use web-application (https://iprp.fimm.fi/). By entering the patient’s clinical information (3 clinical factors) and gene expression profiles (15 genes), the web-tool calculates in real-time the risk points for each factor, along with the patient’s IPRP risk score, the risk category, and the 1-,2-, and 3-year mortality risk. Since the IPRP score is based on the robust gene-pairing approach, there are no pre-requirements on the specific processing of the gene expression input data, and the end-users only need to ensure that the input gene format is consistent across the 15 genes.

### Gene mutation analysis and immune profiling of AML patients

To explore how the IPRP risk groups relate to genetic background of AML patients, we performed mutation analysis in the TCGA-AML and BeatAML cohorts. We focused on the top-20 most frequently mutated genes in these two cohorts and made waterfall plots across the IPRP risk groups (Supplementary Fig. 14A, D). The common finding in both the TCGA-AML and BeatAML cohorts was that TP53 and RUNX1 exhibited significantly higher mutation rates in the IPRP high risk group (*p* < 0.05, Chi-square test). Both *TP53* and *RUNX1* are factors for the ELN2017 adverse risk class. *RUNX1* regulates normal and malignant hematopoiesis, and somatic and germline *RUNX1* mutations are associated with poorer prognosis in AML patients [20], which is consistent with our result. In both of the cohorts, patients in the high risk group had higher TMB levels when considering all somatic mutations (*p* < 0.05, Wilcoxon test; Supplementary Fig. 14B, E), and there was a significant correlation between the TMB levels and IPRP scores (*p* < 0.05, Supplementary Fig. 14C, F).

As an additional immunologic profiling of AML patients, we further carried out immune infiltration analysis using the ssGSAE method to quantify the immune cell content of AML patients, and found that the amount of activated B cells, activated CD4 T cells, activated CD8 T cells, natural killer T cells and type 2 T helper cells in the high risk group were higher than those in the low risk group (adjusted *p* < 0.05, Wilcoxon test and Benjamini-Hochberg correction for multiple testing; Supplementary Fig. 15). This suggests that patients in the high risk group are characterized with a modified immune profile.

### The extended application of IPRP signature into pan-cancer analyses

**Fig. 7:**
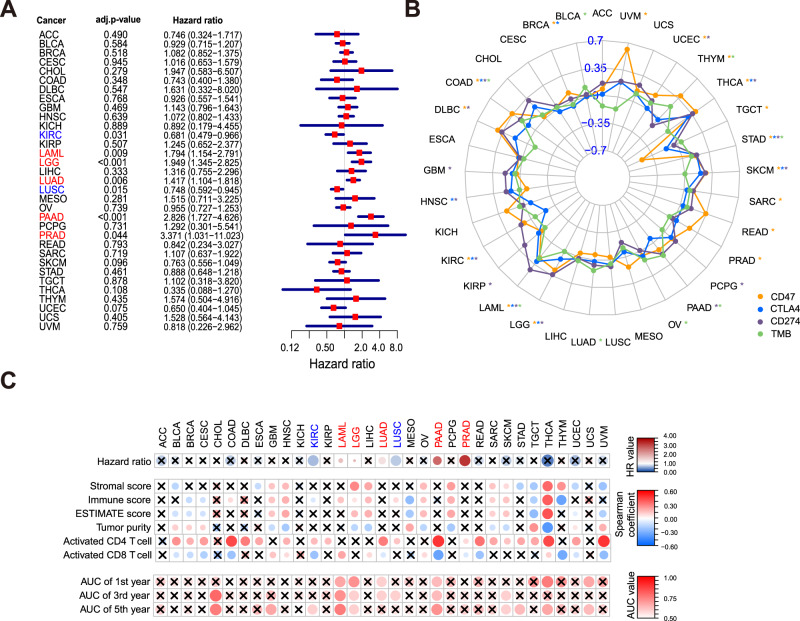
Pan-cancer analysis of the IPRP score. **A** Cox regression analysis of IRPR score across 33 cancer types, where p-values are adjusted with Benjamini and Hochberg method for multiple testing. Red color indicates positive association (HR>1) and blue color negative association (HR < 1). **B** Radar chart of the Spearman correlation between IPRP score and expression of CD47, CTLA4, CD274, and TMB level. The outer side represents a positive correlation, and the inner side represents a negative correlation. The colors mark the immunotherapy targets and the asterisks indicate the statistical significance (*p* < 0.05, Spearman correlation). **C** Tumor microenvironment (TME) and immune infiltration (ssGSEA) analysis, where x indicates no significant association (*p* > 0.05). Statistical significance was assessed with Wald’s test for Hazard ratio for patient prognosis, Spearman correlation for immune-related scoring, and permutation test for ROC-AUC values. ACC Adrenocortical Carcinoma, BLCA Bladder Urothelial Carcinoma, BRCA Breast Invasive Carcinoma, CESC Cervical Squamous Cell carcinoma and Endocervical Adenocarcinoma, CHOL Cholangiocarcinoma, COAD Colon Adenocarcinoma, DLBC Lymphoid Neoplasm Diffuse Large B-cell Lymphoma, ESCA Esophageal Carcinoma, GBM Glioblastoma Multiforme, HNSC Head and Neck Squamous Cell Carcinoma, KICH Kidney Chromophobe, KIRC Kidney Renal Clear Cell Carcinoma, KIRP Kidney Renal Papillary Cell Carcinoma, LAML Acute Myeloid Leukemia, LGG Brain Lower Grade Glioma, LIHC Liver Hepatocellular Carcinoma, LUAD Lung Adenocarcinoma, LUSC Lung Squamous Cell Carcinoma, MESO Mesothelioma, OV Ovarian Serous Cystadenocarcinoma, PAAD Pancreatic Adenocarcinoma, PCPG Pheochromocytoma and Paraganglioma, PRAD Prostate Adenocarcinoma, READ Rectum Adenocarcinoma, SARC Sarcomav, SKCM Skin Cutaneous Melanoma, STAD Stomach Adenocarcinoma, TGCT Testicular Germ Cell Tumors, THCA Thyroid Carcinoma, THYM Thymoma, UCEC Uterine Corpus Endometrial Carcinoma, UCS Uterine Carcinosarcoma, UVM Uveal Melanoma.

Inspired by the results showing that multiple cancer-related pathways shared similar activity profiles with the AML pathway across the prognostic groups (Supplementary Fig. 4), we finally explored the potential wider application of the IPRP signature also in other cancers. More specifically, we performed pan-cancer analysis using the gene expression, point mutation and survival information of 33 cancer patients in TCGA, including 2 hematological cancers and 31 solid tumors. Even though developed in the AML patients, the IPRP score showed a significant prognostic value in 7 cancers types (adjusted *p* < 0.05, Wald’s test and Benjamini-Hochberg correction method; Fig. 7A). The IPRP score was a protective factor in 2 cancers (HR < 1), and a risk factor in 5 cancers (HR > 1), further supporting the varied effect of pyroptosis in different tissues and genetic backgrounds [21]. In comparison with the six established immune subtypes [22], Cox regression identified these immune subtypes as prognostic factors in three cancers, not including AML, but in two additional cancers not captured by the IPRP signature (Supplementary Fig. 16). This shows that the IPRP signature provides complementary and a wider range of pan-cancer application than the established immune subtypes. In total, IPRP distinguished survival differences in patients with 11 tumor types (*p* < 0.05, permutation test; Supplementary Fig. 17).

Even though immunotherapy does not currently play a role in AML treatment, we wanted to explore the relationship between IPRP score and immunotherapy in a pan-cancer analysis, given that the pyroptosis and immunity are both key processes in immunotherapy [23]. When investigating the key immunotherapy targets, the IPRP score and *CD47* expression were significantly correlated in 15 cancer types (*p* < 0.05); only TCGT showed a negative correlation, while the other cancers had positive correlations (Fig. 7B). The IPRP score and *CTLA4* expression were significantly correlated in 9 cancers, among which there were negative correlations in BRCA and KIRC, and the others were all positively correlated (Fig. 7B). Furthermore, the IPRP score was positively correlated with *CD274* (PD-L1) in 14 cancers (Fig. 7B). These results further demonstrate the varying role of IPRP across the cancer types, and show the potential of IPRP score as stratification tool for immunotherapy in the cases where the IPRP score was positively correlated with the expression of immunotherapy targets. In the pan-cancer analysis of TMB, the IPRP score showed positive correlation in 8 cancers (*p* < 0.05; Fig. 7B). Even though the correlation levels remained relatively low, this result supports the use of the IPRP score in a pan-cancer setting to stratify patients for immunotherapy (see Supplementary Data for examples).

To provide potential explanations for the observed differences in the utility and role of the IPRP score across cancer types, we carried out additional analyses of immune infiltration and tumor microenvironment (TME). In the TME analysis (Fig. 7C), we found that the IPRP score and the immune cell content (ImmuneScore) are positively correlated in 12 cancers (*p* < 0.05, including AML), and negatively correlated in 6 cancers (*p* < 0.05, including kidney renal clear cell carcinoma, KIRC). These results indicate that the IPRP score is closely related to the TME status in most cancers, but its effects vary considerably. Along the same lines, the IPRP score and tumor purity were positively correlated in 7 cancers and negatively correlated in 9 cancers. It is known that mainly activated T cells directly kill tumor cells. Consistently, the correlation of the IPRP score was negatively correlated with the content of CD8 + T cells and positively correlated with the CD4 + T cells in KIRC, and the content of CD8 + T cell in the high risk group was greater than that in the low risk group (Supplementary Fig. 18), which is opposite to AML (Supplementary Fig. 15). This can partially explain why the IPRP score is a protective factor in KIRC, while being risk factor in AML (Fig. 7C).

## Discussion

In this retrospective cohort study of AML patients, we established a prognostic IPRP signature and an associated risk score, based on a novel and robust gene pairing approach. Using 5 independent AML cohorts, we demonstrated that the IPRP score leads to more consistent and accurate performance than the existing signatures, and that the IPRP score effectively eliminates batch effects between different patient cohorts and transcriptomic data sets. Our web-tool implementation of the IPRP score and a simple 4-factor nomogram enables an easy application of the risk score for AML patients. Compared to current standards for AML patient stratification, such as age and ELN2017 risk classification, we demonstrated a significant added value of the IPRP risk score for providing improved prognostic prediction for AML patients.

We used AML as a case study to investigate the prognostic power of pyroptosis- and immune-related genes in a hematological malignancy. The treatment responses and overall survival of patients with AML is known to be heterogeneous, influenced both by patient-specific as well as disease-specific factors, making AML a challenging case study for risk scoring and treatment recommendations. Despite the differences in the treatments, age, and genomic background of the AML cohorts, the robust IPRP signature of 10 gene-pairs showed surprisingly consistent performance across the AML cohorts. IPRP was shown to be independent prognostic factor for AML patients, and quite unexpectedly, also in a pan-cancer setting, where it showed association with survival differences and tumor-immune interactions in multiple solid cancer types.

Even though the IPRP score was not designed for immunotherapy response prediction, the analysis of TMB, target expression of immunotherapies, and immune infiltration suggest that patients in the IPRP high risk group tend to be more suitable for immunotherapy. We note these analyses are limited; for instance, PDL1 expression may reflect the proportion of monocytes and lymphocytes in the samples, and hence better predictors of immunotherapy responses will be needed. A diverse range of immunotherapies are now entering clinical development for AML treatment [24], and once treatment data and genome-wide expression and proteomics profiles have been made publicly available, it will be interesting to study the applicability of the current or extended signature for predicting immunotherapy responses of individual patients.

The current work has some limitations and areas worth of future improvement. In the pan-cancer analysis, we used the gene expression levels of the immunotherapy targets as proxy for their protein activity. However, as the mRNA levels do not always correlate with the protein activity, there is a need for further protein-level studies. Transfer learning could be further used to increase the application range of the model also for cancer types without enough training data available [25]. While the current IPRP signature was treatment-agnostic, it was shown to have predictive accuracy for various treatment modalities. In the future, it would be interesting to establish separate models for the main treatment modalities of AML; for instance, predict which AML patients receiving intensive induction treatment become eligible for alloHSCT.

There is a close relationship between pyroptosis and various human diseases, especially malignant tumors [21]. However, as was shown in our pan-cancer results, pyroptosis clearly plays a dual role in the pathogenesis and treatment responses. On one hand, multiple signaling pathways and inflammatory mediators released during pyroptosis are closely implicated in the tumorigenesis and drug resistance. On the other hand, pyroptosis provides means to inhibit the occurrence and development of tumors. Accordingly, the relationship between pyroptosis and cancer biology is complex, and the effects may vary in different tissues and genetic backgrounds. Our results on immune infiltration and TME could only partly explain the differences observed across the cancer types, and more research will be required to illuminate the various effects of pyroptosis on cancer development and treatment.

## Supplementary information

Supplementary Data

Supplementary Tables

Supplementary Figures

## Data Availability

The R codes for implementation of the IPRP score are available at GitHub (https://github.com/kwkx/IPRP). The web-app for the IPRP nomogram is running on the FIMM server (https://iprp.fimm.fi/). The data repositories and accession numbers of the datasets used for developing and testing the IPRP score are listed in Supplementary Data.
