# Supplementary material for: An immunity and pyroptosis gene-pair signature predicts overall survival in acute myeloid leukemia
Source: Leukemia. 2022 Aug 9;36(10):2384–95. doi: 10.1038/s41375-022-01662-6 (PMC9522598; doi:10.1038/s41375-022-01662-6)
Supplement: Supplementary file 1 — Supplementary Data [file 41375_2022_1662_MOESM1_ESM.docx]

**Supplementary Data**

**An immunity and pyroptosis gene-pair signature predicts overall survival in acute myeloid leukemia**

Weikaixin Kong, Liye He, Jie Zhu, Oscar Brück, Kimmo Porkka, Caroline A. Heckman, Sujie Zhu, Tero Aittokallio

## Supplementary Materials/Subjects and Methods

### AML patient cohorts and gene expression data

The patient selection process is shown in Supplementary Figure 1. We used only the diagnostic stage samples and data from the AML patients. Patient samples that lacked survival time or survival status were excluded. Among the 5 AML cohorts, GSE37642^1^ was divided into a training set (n=417) and an internal test set (n=136), which were used for initial signature development and testing, respectively, while the other four data sets were used as independent validation sets: TCGA-AML (n=140)^2^, GSE106291 (n=250)^3^, BeatAML (n=290)^4^ and FIMM AML (n=94)^5^. The TCGA expression, clinical and mutation data were downloaded from the GDC Data Portal (https://portal.gdc.cancer.gov/), and the data of studies (GSE37642 and GSE106291) were obtained from the GEO database (<https://www.ncbi.nlm.nih.gov/gds>). The clinical, survival, expression and mutation data of patients in the BeatAML cohort were obtained from Tyner et al^4^. Among these AML cohorts, GSE37642 used microarray platform, and GSE106291, BeatAML and FIMM AML profiled the patients with genome-wide RNA-seq, whereas TCGA AML cohort contained both RNA-seq and microarray data. In the GSE37642 cohort, we used expression data from the HGU-133plus2 array (n=140) and HGU-133A array (n=422), among which 9 patients did not have OS data, hence leading to a total of 553 patients for the analyses. The RNA-seq data of FIMM AML was profiled as described before^6^.

### Clustering of AML patients in the training set

For clustering of the AML samples, we used the expression of genes related both to the pyroptosis and immune process of AML pathophysiology. Based on the previous studies^7-9^, we extracted 33 genes involved in cell pyroptosis: *GPX4, NLRP7, NLRP2, CASP3, CASP6, TNF, IL1B, IL18,* *CASP8, NLRP6, IL6, GSDMA, GSDMC, PYCARD, CASP5, AIM2, NOD2, NLRC4, NLRP3, CASP4, CASP1, PRKACA, ELANE, TIRAP, SCAF11, PJVK, CASP9, NOD1, PLCG1, NLRP1, GSDME, GSDMD, GSDMB*. These 33 genes were subjected to univariate survival analysis in the training AML set (see above), and the expression levels of the 22 genes that distinguished the survival status of patients (p<0.05, Log Rank test) were used to cluster the AML patients in the training set using hierarchical clustering with Euclidean distance. The p-values from univariate survival analysis were adjusted with Benjamini-Hochberg method. The area under the cumulative distribution function (CDF) curve was analyzed visually to find the optimal number *k* of clusters. The above analyses were carried out using the "limma", "survival", "survminer", and "ConsensusClusterPlus" packages in R.

We additionally clustered the patients by immune-related genes. Based on the Immport database (https://www.immport.org/shared/), we obtained the list of 2483 immune-related genes, and used 1047 genes without missing expression data in the 6 AML data sets. In the end, we obtained 156 immune-related genes that distinguished the survival status of the AML patients in the training data (p<0.05, Log Rank test; Benjamini-Hochberg correction). There were no overlapping genes between the 156 immune-related and 22 pyroptosis-related genes, which ensures the orthogonality of the two cluster analyses, carried out similarly as described above. Through the two cluster analysis solutions, we finally divided the AML patients in the training set into 4 groups: double favorable prognosis group (FF), double unfavorable prognosis group (UU), pyroptosis favorable and immune unfavorable group (FU), pyroptosis unfavorable and immune favorable group (UF) (Supplementary Figure 2).

### Pathway analysis of gene signatures

To explore the transcriptomic changes between the FF and UU groups of AML patients, we used gene set variation analysis (GSVA)^10^ to assess the degree of enrichment of the KEGG pathways. The limma R-package was used to find differentially-enriched pathways, where we further visualized the 20 pathways with statistically significant fold change (FC) difference (|log_2_FC|>1 and FDR<0.05). The above analyses were carried out additionally using the "GSVA" and "pheatmap" packages in R.

### Benchmarking of the prognostic model

The unpaired IPRP signature was constructed based on the 26 genes selected by the first LASSO regression, subjected to multivariate Cox regression, to study the effect of the pairing procedure (Supplementary Figure 2). The unpaired IPRP signature contained 9 genes, of which 5 genes were risk factors and 4 genes were protective factors (Supplementary Table 4). The pyroptosis-only signature was constructed based on the 22 pyroptosis-related genes, subjected to multivariate Cox regression, to study how much the pyroptosis-related genes contribute to the prognostic prediction. Pyroptosis signature contained 6 genes, all of which were protective factors (Supplementary Table 5). The IRG signature is a prognostic model established for immune-related genes in AML patients^11^. Autophagy and Hypoxia signatures were extracted from AML patients comprising of genes related to autophagy and hypoxia, respectively, and which have shown to have good prognostic value^12, 13^. CXCR signature comprises of genes of the chemokine receptor family which plays an important role in regulating the differentiation and development of memory cells and effector T cells^14^. The 24-gene signature was established by collecting multiple AML datasets and signatures before 2013 (note: our training set is among these studies), and the prognostic model was constructed by meta-analysis^1^. LSC17 and pLSC6 signatures were constructed based on prognostic biomarkers related to stemness in AML^15, 16^. The 7-gene and PS29MRC signatures were established based on DNA methylation-related genes^17^ and therapy resistance related genes^3^, respectively.

To benchmark our paired IPRP signature, we compared its performance against these 10 signatures in the internal test set and four independent validation sets of AML patients (see section AML patient cohorts and gene expression data above). First, we analyzed the distributions of the risk scores, with the assumption that if the score distributions across different patient cohorts and data sets are similar, we can regard the prognostic model stable in various data types and patient cohorts, hence being robust against batch effects. Then, we further calculated the area under the receiver operating characteristic (AUC-ROC) curve for predicting the overall survival (OS) time of the patients. The 1-, 2-, and 3-year AUC-ROC values were used to evaluate the accuracy of prognostic models. Permutation test with 300 permuted datasets was used to evaluate significance of AUC-ROCs. In the permutation test, the relationship between patient survival and IPRP score was randomly permuted 300 times, and the prognostic prediction was made using the model, resulting in empirical distribution of AUC-ROC values under the null hypothesis (i.e., null distribution). We considered models with a permutation p<0.05 significantly better than a random classifier (AUC-ROC=0.5). For comparison between different signatures, we used non-parametric paired Wilcoxon test.

### Establishment of a nomogram for AML patients

To explore whether the IPRP signature is an independent or additional risk factor among other molecular and clinical features, we conducted a univariate and multivariate independent prognostic analysis in the BeatAML data set (n=224), which contains various types of molecular and clinical information. The BeatAML cohort was divided into training set (n=152) and test set (n=72). In addition, the TCGA AML cohort (n=139) and FIMM AML cohort (n=94) were also used for validation of the nomogram. The training set was used initially for independent prognostic analysis and to establish a nomogram among the independent risk factors with multivariate Cox regression. The performance of the nomogram was then confirmed in each of the three test sets.

We used the following criteria for selecting variables in the construction of nomogram: (i) The proportion of missing values across the patients for a particular variable in the BeatAML dataset must be less than 10%. (ii) The proportion of each class of categorical variables must be greater than 3% in the training set. (iii) The variables included into the multivariate Cox regression modelling must be obtained at the diagnosis stage of patients. (iv) The selected variables included into the nomogram must exist also in the TCGA AML and FIMM AML cohorts to enable wider testing.

To make this nomogram available for other researchers, including those without programming skills, it was deployed on the FIMM server through the "shiny" R package (<https://iprp.fimm.fi/>).

### Gene mutation and immune profile analyses in AML cohorts

The Chi-square test and Wilcoxon test were used to compare the gene mutations and TMB values, respectively, between the IPRP high risk and low risk groups. Spearman rank correlation was used to quantify the association between the TMB levels and IPRP risk scores among the AML patients. The significance of the correlation coefficient was assessed with the Ryser's formula as described before^18^. The rank correlation analyses were carried out using the spearman R-package. Single-sample Gene Set Enrichment Analysis^19^ (ssGSEA) was used to calculate the differences in the immune cell contents between the high risk and the low risk groups. ssGSEA is a deconvolution algorithm based on bulk gene expression profiles, which accurately quantifies the content of immune cells in the cancer tissue^20^. In this process, we used the limma, GSEABase, ggpubr and reshape2 packages in R.

**Pan-cancer analysis of overall survival and tumor immunity**

Previously, Thorsson et al. established six immune subtypes that affect tumor-immune interactions across cancer types and which are closely related to cancer prognosis^21^. To compare the result with our IPRP signature, we performed Cox regression of these six immune subtypes in the pan-cancer setting. In addition, we also evaluated the IPRP signature using the AUC-ROC analyses across the 33 different cancers. We used a permutation test to assess whether the ROC-AUC values are significant by performing 300 random permutations of the data.

To explore the relationship between IPRP score and tumor microenvironment (TME) in 33 cancers, the ESTIMATE algorithm was used to calculate the TME score for each cancer patient, including StromalScore, ImmuneScore, ESTIMATEScore, and TumorPurity^22^. StromalScore represents the content of stromal cells in the tissue, ImmuneScore the content of immune cells, ESTIMATEScore the sum of the content of immune cells and stromal cells, and TumorPurity represents the content of tumor cells. By calculating the Pearson correlation coefficient between the IPRP score and the four types of TME scores, we tested how well the IPRP score associates with the immune microenvironment status among the 33 cancers. In addition, we also used the ssGSEA method^19^ to calculate the Spearman correlation coefficient between the IPRP score and activated T cell content in the pan-cancer analyses. In the above analysis, we used the "limma", "GSEABase" "estimate" and "corrplot" package in R.

### Pan-cancer analysis of IPRP score and immunotherapy responses

To test how well the IPRP score predicts the immunotherapy response in solid tumors, we used five immunotherapy cohorts, namely GSE78220 (melanoma, n=28, anti-PD1)^23^, GSE67501 (renal cell carcinoma, n=11, anti-PD1)^24^, the data from Kim et al.^25^ (gastric cancer, n=45, anti-PD1), the data from Lauss et al.^26^ (melanoma, n=25, anti-PD1) and IMvigor cohort data (urothelial cancer, n=299, anti-PDL1). The data of GSE78220 and GSDE67501 were downloaded from the GEO database and the data of IMvigor cohort were obtained using the "IMvigor210CoreBiologies" package in R. Using the Wilcoxon test, we tested the relationship between the IPRP score and immunotherapy response in these five patient cohorts. In addition, we also carried out survival analyses based on the IPRP risk groups to explore the prognosis classification of patients after anti-PDL1 and anti-PD1 treatments in the two cohorts, IMvigor and GSE788220, which have complete OS data available.

**Supplementary Results**

**Evaluation of the influence of batch effects and treatment modalities**

To evaluate the sensitivity of IPRP score and other signatures to batch effects, we combined the expression data from the 5 test cohorts, and then made prognostic predictions in the combined test set (Supplementary Figure 7), with or without using the ComBat function to eliminate batch effects^27^. Before using ComBat, samples from the 5 test sets were grouped according to the cohorts (Supplementary Figure 7A), whereas the ComBat normalized the expression profiles relatively similar across the cohorts (Supplementary Figure 7B). Importantly, regardless whether applying ComBat or not, IPRP score showed accurate predictions across the cohorts (Supplementary Figure 7C), which further indicates that the paired IPRP signature was less influenced by the batch effects from the patient cohorts, when compared to the other signatures. To further explore robustness of the IPRP score across different transcriptomic platforms, we used the paired TCGA-AML microarray and RNA-seq data sets to calculate risk score for each sample using the two technologies. Among the signatures, IPRP score showed the highest correlation (R=0.77, p<0.001, Supplementary Figure 8), which further proves its robustness and wide applicability.

Finally, we evaluated the accuracy of the IPRP score in AML patients who received different treatment regimens in the BeatAML and FIMM AML cohorts, where detailed patient-level treatment information was available (Supplementary Table 1). We divided the patients into two broad treatment groups according to whether or not the patient received curative and/or prolonging treatment. In the BeatAML cohort, IPRP distinguished the survival differences in both of the treatment groups (p<0.05, log rank test; Supplementary Figure 9 A,B). In the FIMM AML cohort, the survival difference in the other treatment group was not significant, most likely due to the limited number of patients receiving palliative care or unknown treatment type (n=11). Based on the ROC analyses, IPRP score showed consistent prognostic accuracy between the two cohorts (p<0.05, permutation test, Supplementary Figure 10). These results demonstrate that the IPRP score predicts survival differences accurately and robustly for various treatment types and using different transcriptomic platforms.

### Pan-cancer analysis of IPRP score and immunotherapy responses

To investigate whether the IPRP score could be used as an immunotherapy response predictor in solid tumors, we searched for cohorts with genome-wide gene expression and clinical response information available for patients treated with immunotherapies. We collected 5 such cohorts and found that in the IMvigor cohort, the patients with urothelial cancer who responded to anti-PDL1 therapy showed also a higher IPRP score (adjusted p=0.048, Wilcoxon test and Benjamini-Hochberg method for multiple testing correction; Supplementary Figure 19A). Since only IMvigor and GSE78220 cohorts contained complete OS data, we carried out survival analysis in these two groups, where it was observed that the IPRP risk groups were associated with the survival differences in both of these two cohorts (adjusted p<0.05, Log Rank test; Supplementary Figure 20A, B). As expected, patients in the high risk group showed better survival status, which indicates that these patients were more likely to respond to the immunotherapy. In the other immunotherapy cohorts, we observed varied results (Supplementary Figure 19B-E), which is likely due to relatively small sample sizes of the treated cohorts, as well as because of the complex interplay between the cancer types and immunotherapy modalities.

**References**

1. Li Z, Herold T, He C, Valk PJ, Chen P, Jurinovic V*, et al.* Identification of a 24-gene prognostic signature that improves the European LeukemiaNet risk classification of acute myeloid leukemia: an international collaborative study. *Journal of clinical oncology : official journal of the American Society of Clinical Oncology* 2013 Mar 20; **31**(9)**:** 1172-1181.

2. Ley TJ, Miller C, Ding L, Raphael BJ, Mungall AJ, Robertson A*, et al.* Genomic and epigenomic landscapes of adult de novo acute myeloid leukemia. *The New England journal of medicine* 2013 May 30; **368**(22)**:** 2059-2074.

3. Herold T, Jurinovic V, Batcha AMN, Bamopoulos SA, Rothenberg-Thurley M, Ksienzyk B*, et al.* A 29-gene and cytogenetic score for the prediction of resistance to induction treatment in acute myeloid leukemia. *Haematologica* 2018 Mar; **103**(3)**:** 456-465.

4. Tyner JW, Tognon CE, Bottomly D, Wilmot B, Kurtz SE, Savage SL*, et al.* Functional genomic landscape of acute myeloid leukaemia. *Nature* 2018 Oct; **562**(7728)**:** 526-531.

5. Malani D, Kumar A, Brück O, Kontro M, Yadav B, Hellesøy M*, et al.* Implementing a Functional Precision Medicine Tumor Board for Acute Myeloid Leukemia. *Cancer discovery* 2022 Feb; **12**(2)**:** 388-401.

6. Kumar A, Kankainen M, Parsons A, Kallioniemi O, Mattila P, Heckman CA. The impact of RNA sequence library construction protocols on transcriptomic profiling of leukemia. *BMC genomics* 2017 Aug 17; **18**(1)**:** 629.

7. Karki R, Kanneganti TD. Diverging inflammasome signals in tumorigenesis and potential targeting. *Nature reviews Cancer* 2019 Apr; **19**(4)**:** 197-214.

8. Xia X, Wang X, Cheng Z, Qin W, Lei L, Jiang J*, et al.* The role of pyroptosis in cancer: pro-cancer or pro-"host"? *Cell death & disease* 2019 Sep 9; **10**(9)**:** 650.

9. Wang B, Yin Q. AIM2 inflammasome activation and regulation: A structural perspective. *Journal of structural biology* 2017 Dec; **200**(3)**:** 279-282.

10. Hänzelmann S, Castelo R, Guinney J. GSVA: gene set variation analysis for microarray and RNA-seq data. *BMC bioinformatics* 2013 Jan 16; **14:** 7.

11. Zhu R, Tao H, Lin W, Tang L, Hu Y. Identification of an Immune-Related Gene Signature Based on Immunogenomic Landscape Analysis to Predict the Prognosis of Adult Acute Myeloid Leukemia Patients. *Frontiers in oncology* 2020; **10:** 574939.

12. Jiang F, Mao Y, Lu B, Zhou G, Wang J. A hypoxia risk signature for the tumor immune microenvironment evaluation and prognosis prediction in acute myeloid leukemia. *Scientific reports* 2021 Jul 19; **11**(1)**:** 14657.

13. Fu D, Zhang B, Wu S, Zhang Y, Xie J, Ning W*, et al.* Prognosis and Characterization of Immune Microenvironment in Acute Myeloid Leukemia Through Identification of an Autophagy-Related Signature. *Frontiers in immunology* 2021; **12:** 695865.

14. Lu C, Zhu J, Chen X, Hu Y, Xie W, Yao J*, et al.* Risk Stratification in Acute Myeloid Leukemia Using CXCR Gene Signatures: A Bioinformatics Analysis. *Frontiers in oncology* 2020; **10:** 584766.

15. Ng SW, Mitchell A, Kennedy JA, Chen WC, McLeod J, Ibrahimova N*, et al.* A 17-gene stemness score for rapid determination of risk in acute leukaemia. *Nature* 2016 Dec 15; **540**(7633)**:** 433-437.

16. Elsayed AH, Rafiee R, Cao X, Raimondi S, Downing JR, Ribeiro R*, et al.* A six-gene leukemic stem cell score identifies high risk pediatric acute myeloid leukemia. *Leukemia* 2020; **34**(3)**:** 735-745.

17. Marcucci G, Yan P, Maharry K, Frankhouser D, Nicolet D, Metzeler KH*, et al.* Epigenetics meets genetics in acute myeloid leukemia: clinical impact of a novel seven-gene score. *Journal of clinical oncology : official journal of the American Society of Clinical Oncology* 2014 Feb 20; **32**(6)**:** 548-556.

18. van de Wiel MA, Bucchianico AD. Fast computation of the exact null distribution of Spearman's ρ and Page's L statistic for samples with and without ties. *Journal of Statistical Planning and Inference* 2001 2001/01/01/; **92**(1)**:** 133-145.

19. Barbie DA, Tamayo P, Boehm JS, Kim SY, Moody SE, Dunn IF*, et al.* Systematic RNA interference reveals that oncogenic KRAS-driven cancers require TBK1. *Nature* 2009 Nov 5; **462**(7269)**:** 108-112.

20. Huo J, Wu L, Zang Y. Identification and validation of a novel immune-related signature associated with macrophages and CD8 T cell infiltration predicting overall survival for hepatocellular carcinoma. *BMC medical genomics* 2021 Sep 20; **14**(1)**:** 232.

21. Thorsson V, Gibbs DL, Brown SD, Wolf D, Bortone DS, Ou Yang TH*, et al.* The Immune Landscape of Cancer. *Immunity* 2019 Aug 20; **51**(2)**:** 411-412.

22. Yoshihara K, Shahmoradgoli M, Martínez E, Vegesna R, Kim H, Torres-Garcia W*, et al.* Inferring tumour purity and stromal and immune cell admixture from expression data. *Nature communications* 2013; **4:** 2612.

23. Hugo W, Zaretsky JM, Sun L, Song C, Moreno BH, Hu-Lieskovan S*, et al.* Genomic and Transcriptomic Features of Response to Anti-PD-1 Therapy in Metastatic Melanoma. *Cell* 2016 Mar 24; **165**(1)**:** 35-44.

24. Ascierto ML, McMiller TL, Berger AE, Danilova L, Anders RA, Netto GJ*, et al.* The Intratumoral Balance between Metabolic and Immunologic Gene Expression Is Associated with Anti-PD-1 Response in Patients with Renal Cell Carcinoma. *Cancer immunology research* 2016 Sep 2; **4**(9)**:** 726-733.

25. Kim ST, Cristescu R, Bass AJ, Kim KM, Odegaard JI, Kim K*, et al.* Comprehensive molecular characterization of clinical responses to PD-1 inhibition in metastatic gastric cancer. *Nature medicine* 2018 Sep; **24**(9)**:** 1449-1458.

26. Lauss M, Donia M, Harbst K, Andersen R, Mitra S, Rosengren F*, et al.* Mutational and putative neoantigen load predict clinical benefit of adoptive T cell therapy in melanoma. *Nature communications* 2017 Nov 23; **8**(1)**:** 1738.

27. Johnson WE, Li C, Rabinovic A. Adjusting batch effects in microarray expression data using empirical Bayes methods. *Biostatistics (Oxford, England)* 2007 Jan; **8**(1)**:** 118-127.
