# Supplementary material for: An immunity and pyroptosis gene-pair signature predicts overall survival in acute myeloid leukemia
Source: Leukemia. 2022 Aug 9;36(10):2384–95. doi: 10.1038/s41375-022-01662-6 (PMC9522598; doi:10.1038/s41375-022-01662-6)
Supplement: Supplementary file 3 — Supplementary Figures [file 41375_2022_1662_MOESM3_ESM.pdf]

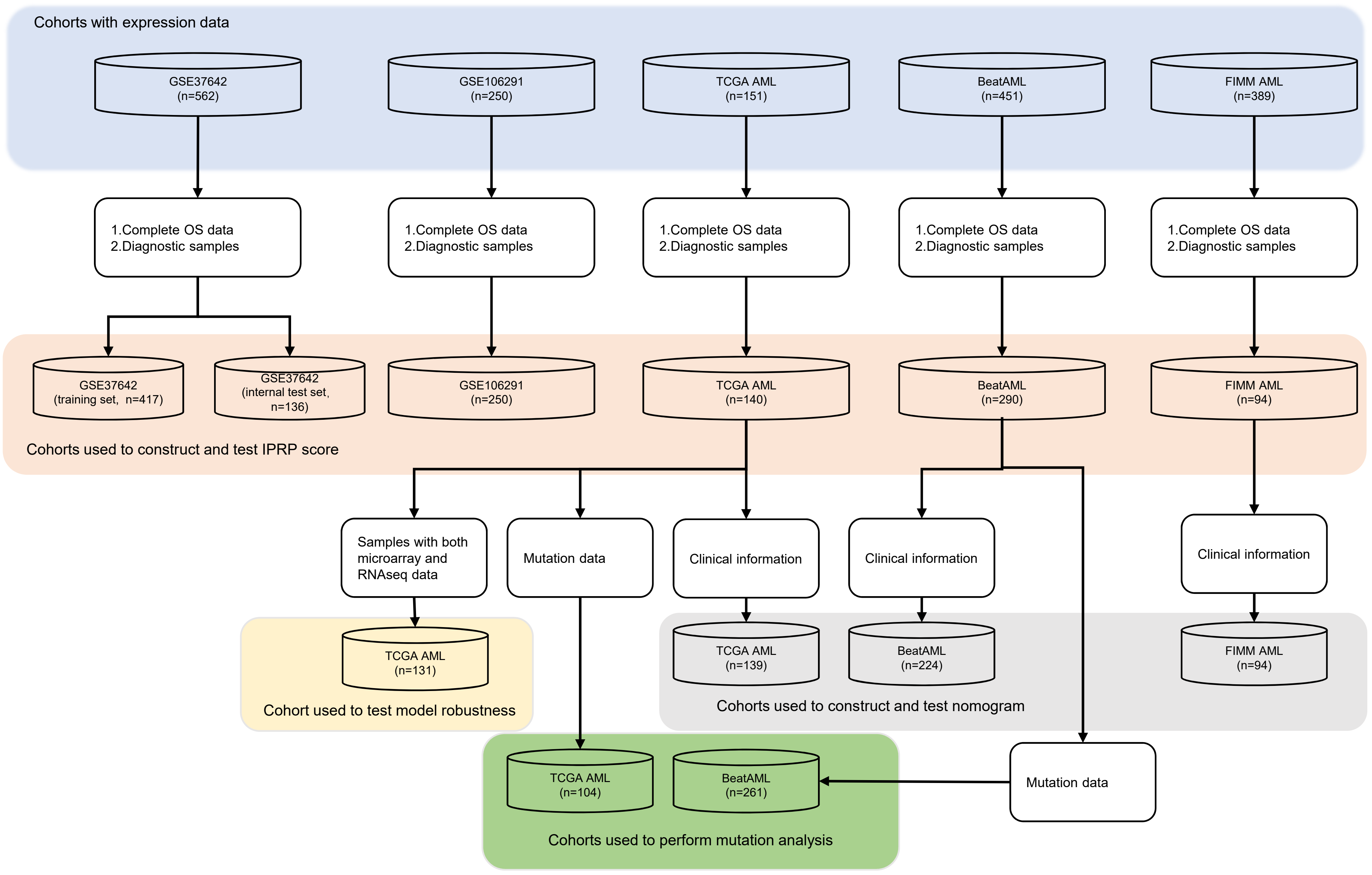

Supplementary Figure 1. The consort flow diagram for patient selection in the AML cohorts.

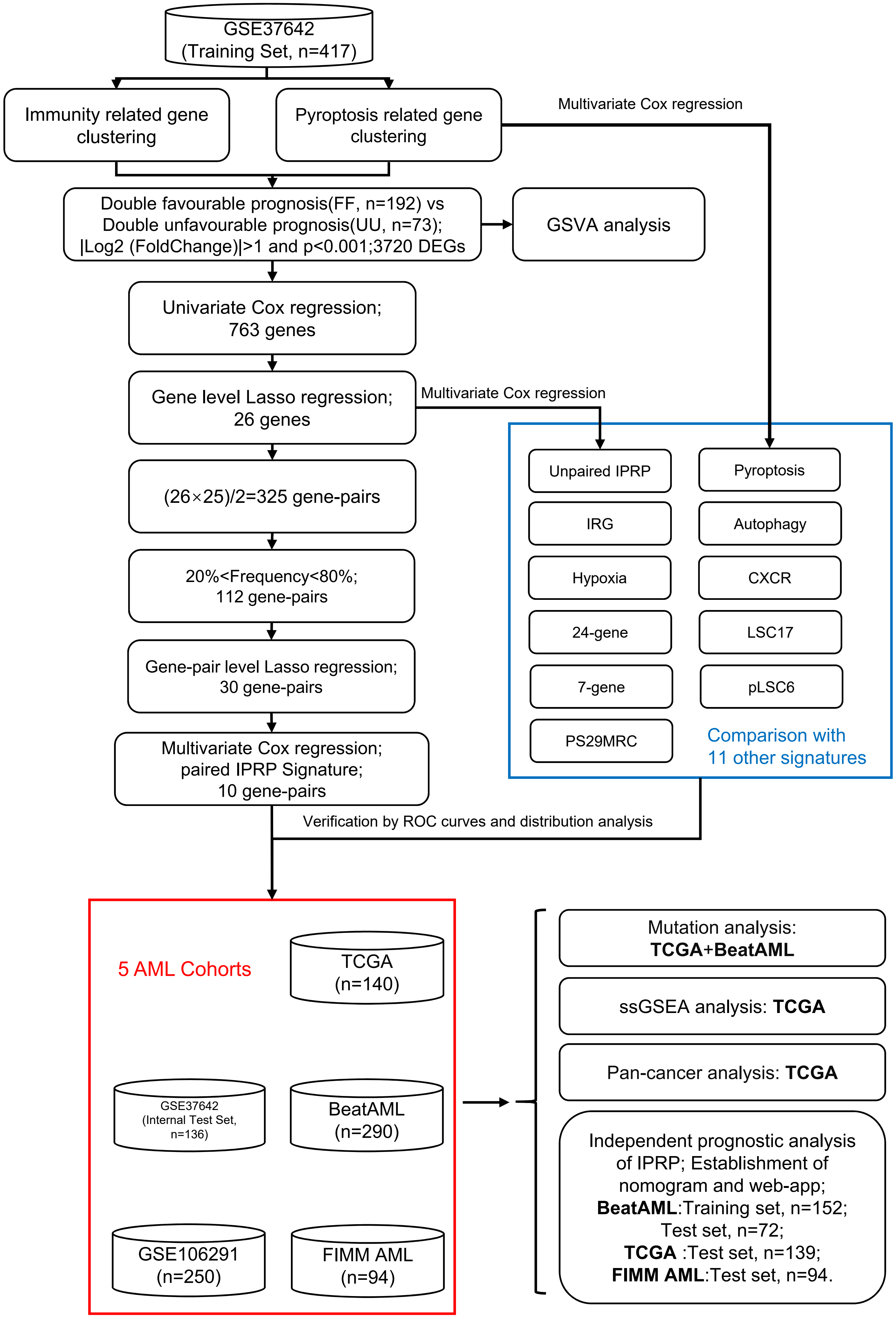

Supplementary Figure 2. Flow chart of the construction and validation of the IPRP signature.

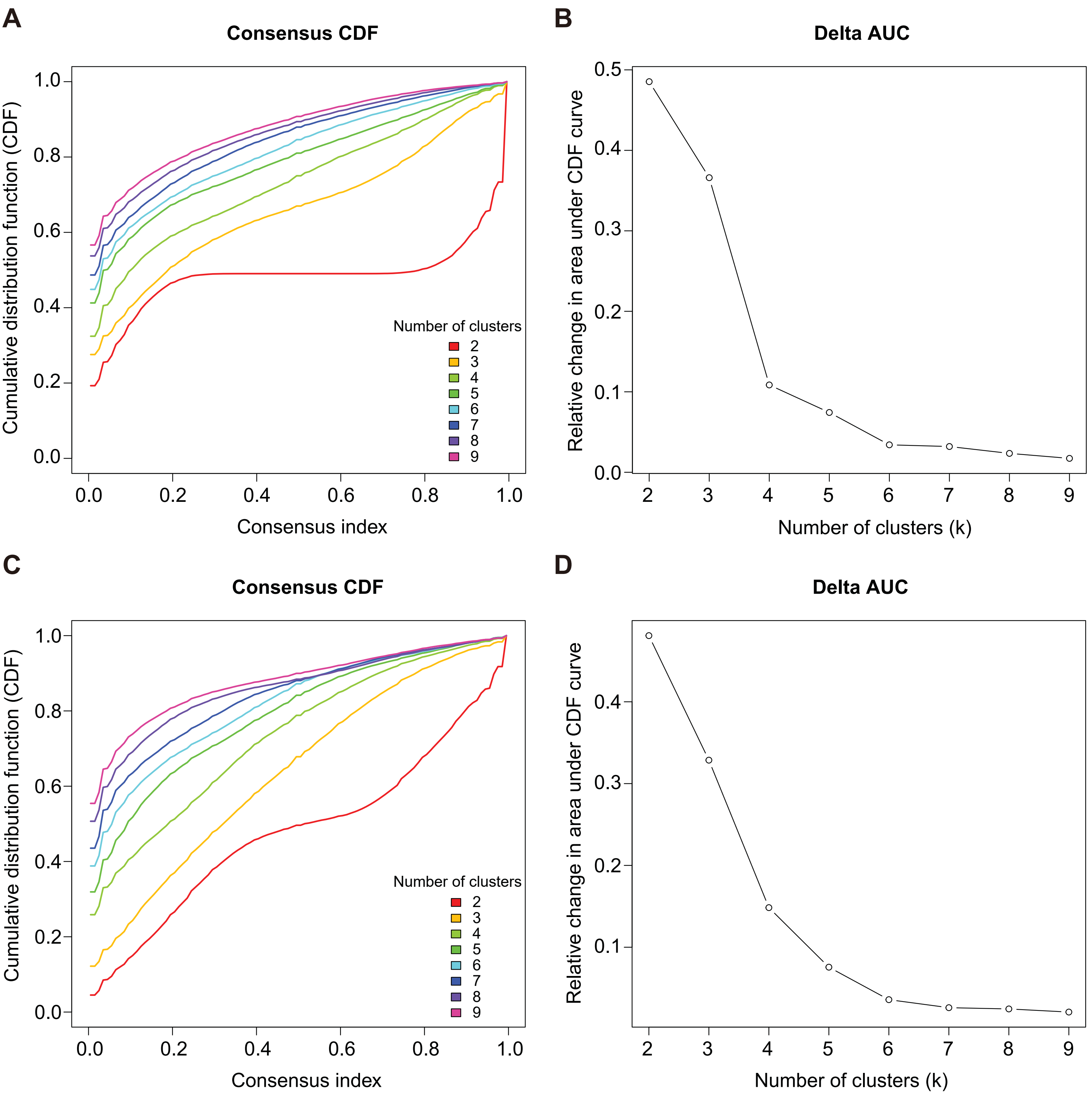

Supplementary Figure 3. The selection of the number of clusters, k, when clustering AML patients based on their gene expression profiles in the training set. (A) Consensus CDF curves in cluster analysis based on the pyroptosis-related genes. (B) Relative AUC changes as a function of k in pyroptosis-related cluster analysis. (C) CDF curves in cluster analysis related to immunity. (D) Relative AUC changes as a function of k in immunity-related cluster analysis.

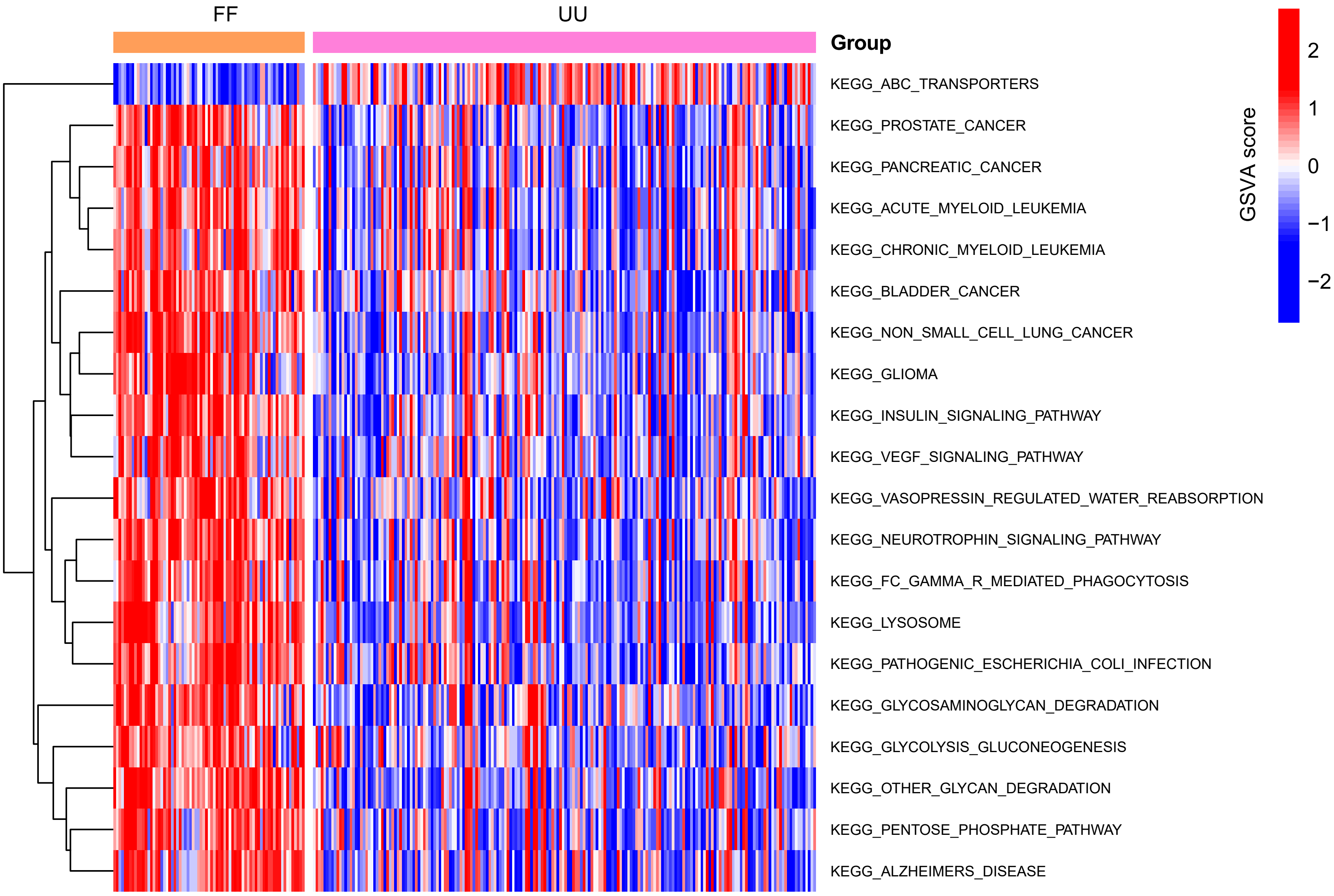

Supplementary Figure 4. The enriched KEGG pathways in the GSVA analysis using the 3720 differentially expressed genes between the FF and UU prognostic groups in the training set.

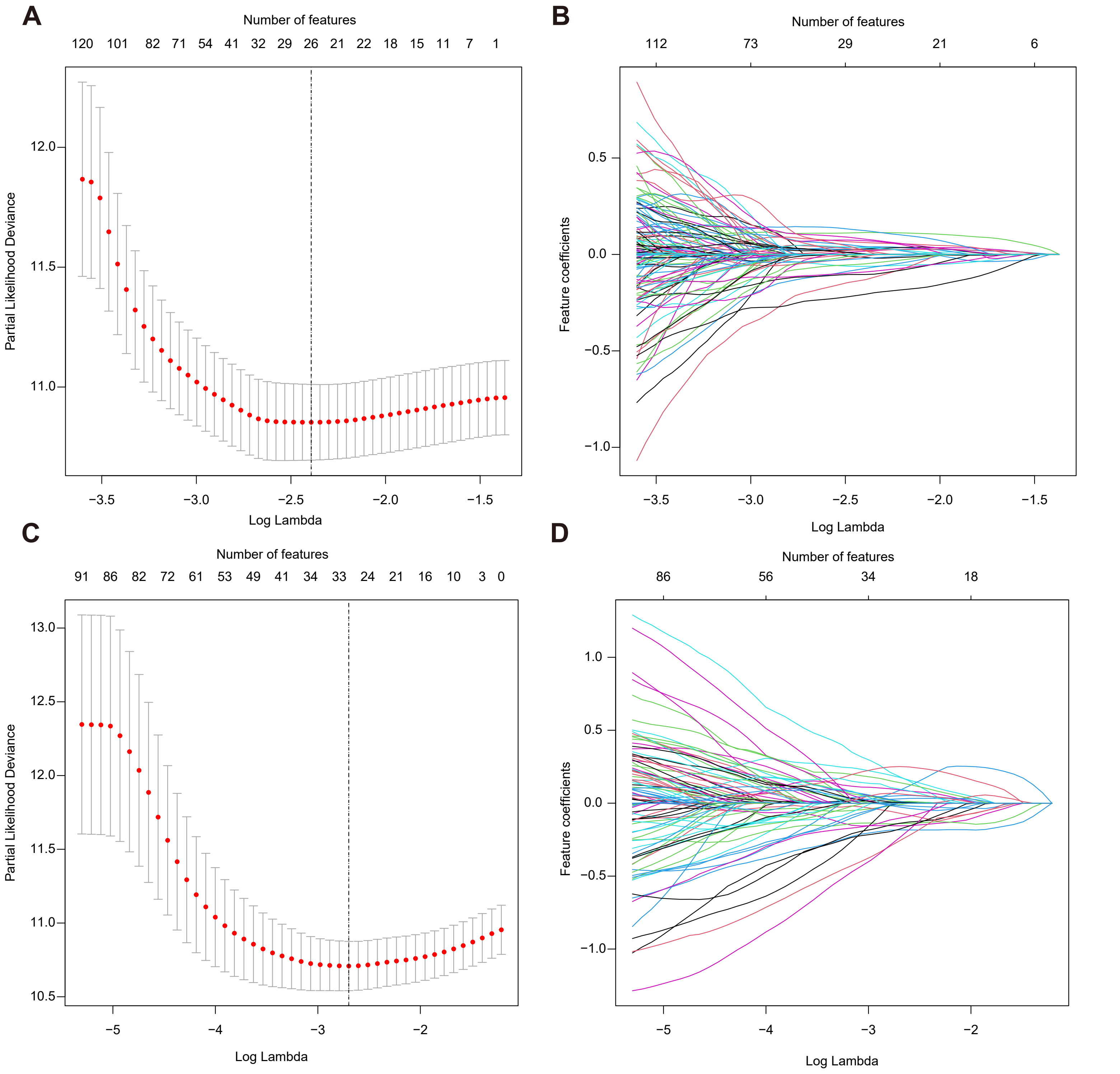

Supplementary Figure 5. The selection of survival predictive genes (A, B) and gene-pairs (C, D) using LASSO regression in the training set. (A) The partial likelihood deviance in cross-validation (CV) as a function of the penalty coefficient lambda. The dotted line shows the lambda value of 0.09 at the minimum partial likelihood deviance level, suggesting 26 genes as optimal predictive features. Standard errors are calculated over 1000 CV rounds. (B) The coefficients of the 26 genes as a function of the penalty coefficient (lambda). (C) The partial likelihood deviance in cross-validation (CV) as a function of the penalty coefficient lambda values. The dotted line shows the lambda value 0.07 at the minimum partial likelihood deviance level, suggesting 30 gene-pairs as optimal predictive features. Standard errors are calculated over 1000 CV rounds. (D) The coefficient of the 30 gene-pairs as a function of the penalty coefficient lambda values.

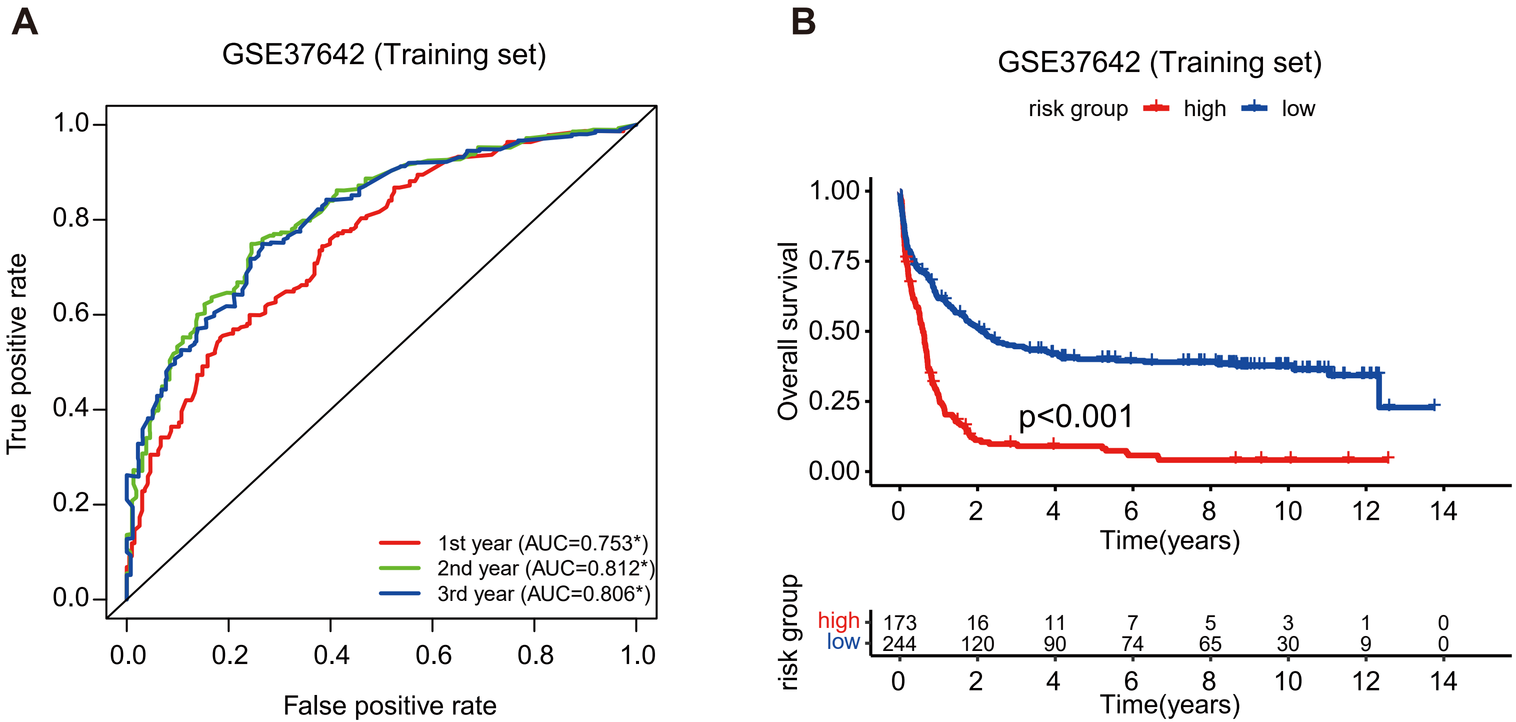

Supplementary Figure 6. Prognostic prediction in the AML training set. (A) ROC curves for prognostic prediction, where significance of ROC-AUC values was assessed with permutation test. (B) Survival curves, where survival differences were assessed with log-rank test.

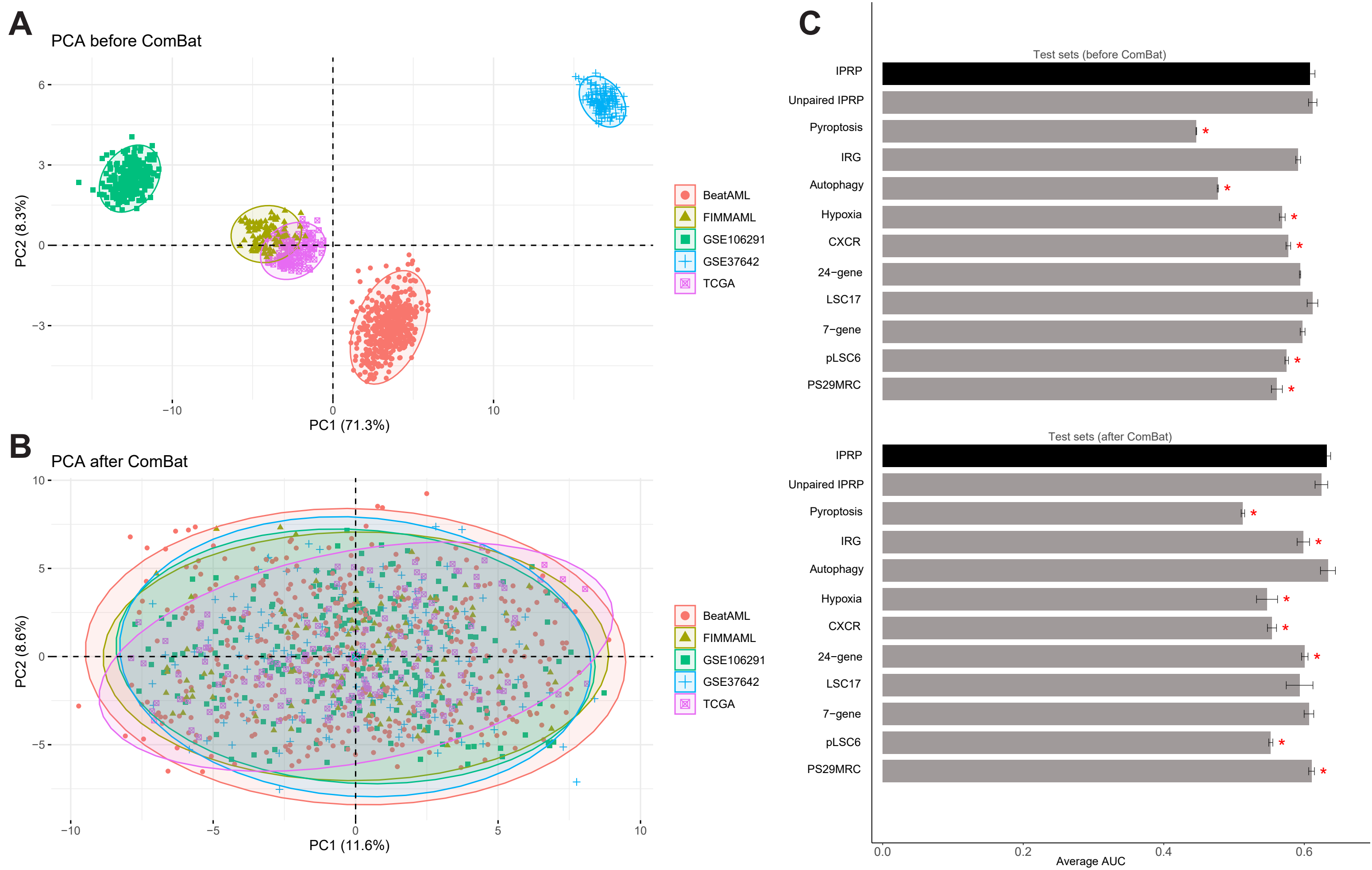

Supplementary Figure 7. Principal component analysis (PCA) of genome-wide gene expression profiles (A) before and (B) after the ComBat batch correction across the test cohorts. (C) Accuracy of the IPRP score and the other 11 risk scores in the combined test set (the bars indicate average ROC-AUC and SD over the 5 test sets). The upper panel shows the prediction accuracy when combining patients from the 5 test cohorts together using the original gene expression profiles, whereas in the lower panel the gene expression profiles of patients from the 5 test cohorts are combined using the ComBat function. Red * indicates that the ROC-AUC is significantly lower than that of IPRP signature using paired Wilcoxon test ($p<0.05$).

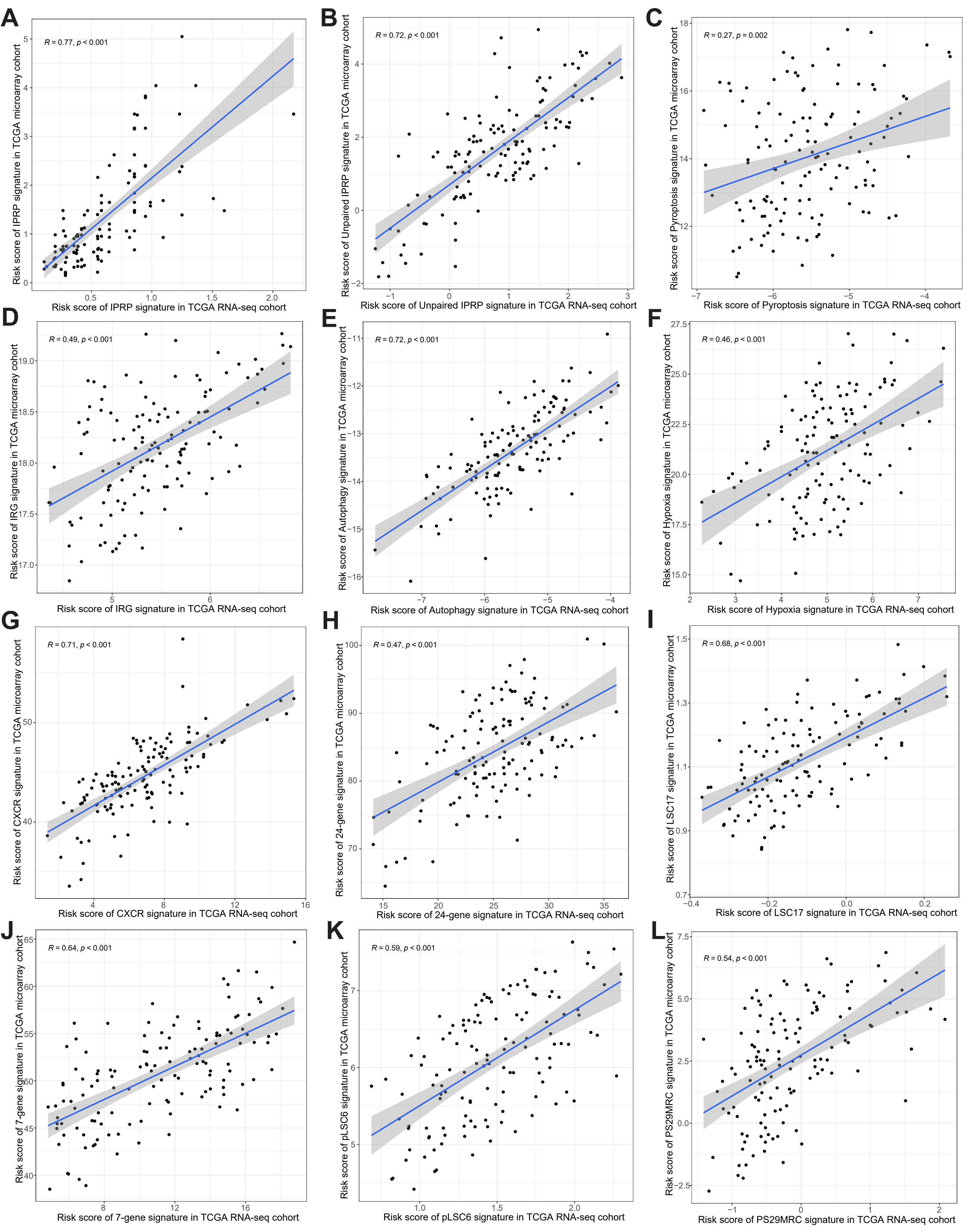

Supplementary Figure 8. The relationships of the risk scores between TCGA-AML RNA-seq and microarray data using Spearman coefficient. (A) IPRP signature. (B) Unpaired IPRP signature. (C) Pyroptosis signature. (D) IRG signature. (E) Autophagy signature. (F) Hypoxia signature. (G) CXCR signature. (H) 24-gene signature. (I) LSC17 signature. (J) 7-gene signature. (K) pLSC6 signature. (L) PS29MRC signature. Points indicate individual patient samples in the TCGA-AML cohort (n=131).

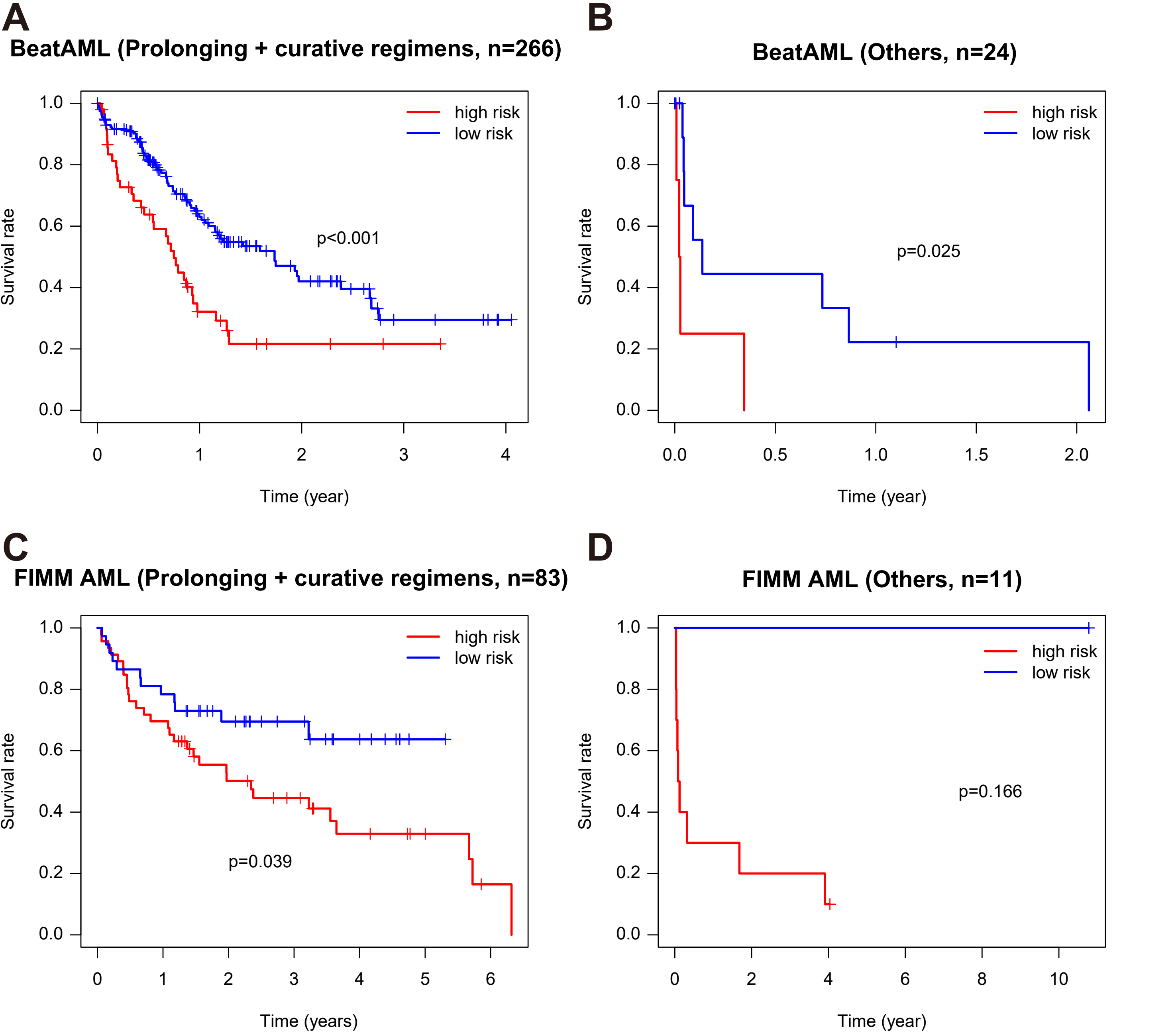

Supplementary Figure 9. Survival curves of patients in the IPRP high and low-risk groups receiving different treatment modalities. (A) The prolonging and curative treatment in the BeatAML cohort. (B) Other treatment modalities (palliative care or unknown type) in the BeatAML cohort. (C) The prolonging and curative treatment in the FIMM AML cohort. (D) Other treatment modalities (palliative care or unknown type) in the FIMM AML cohort. Statistical significance of the survival differences between the two treatment groups was assessed with Log Rank test.

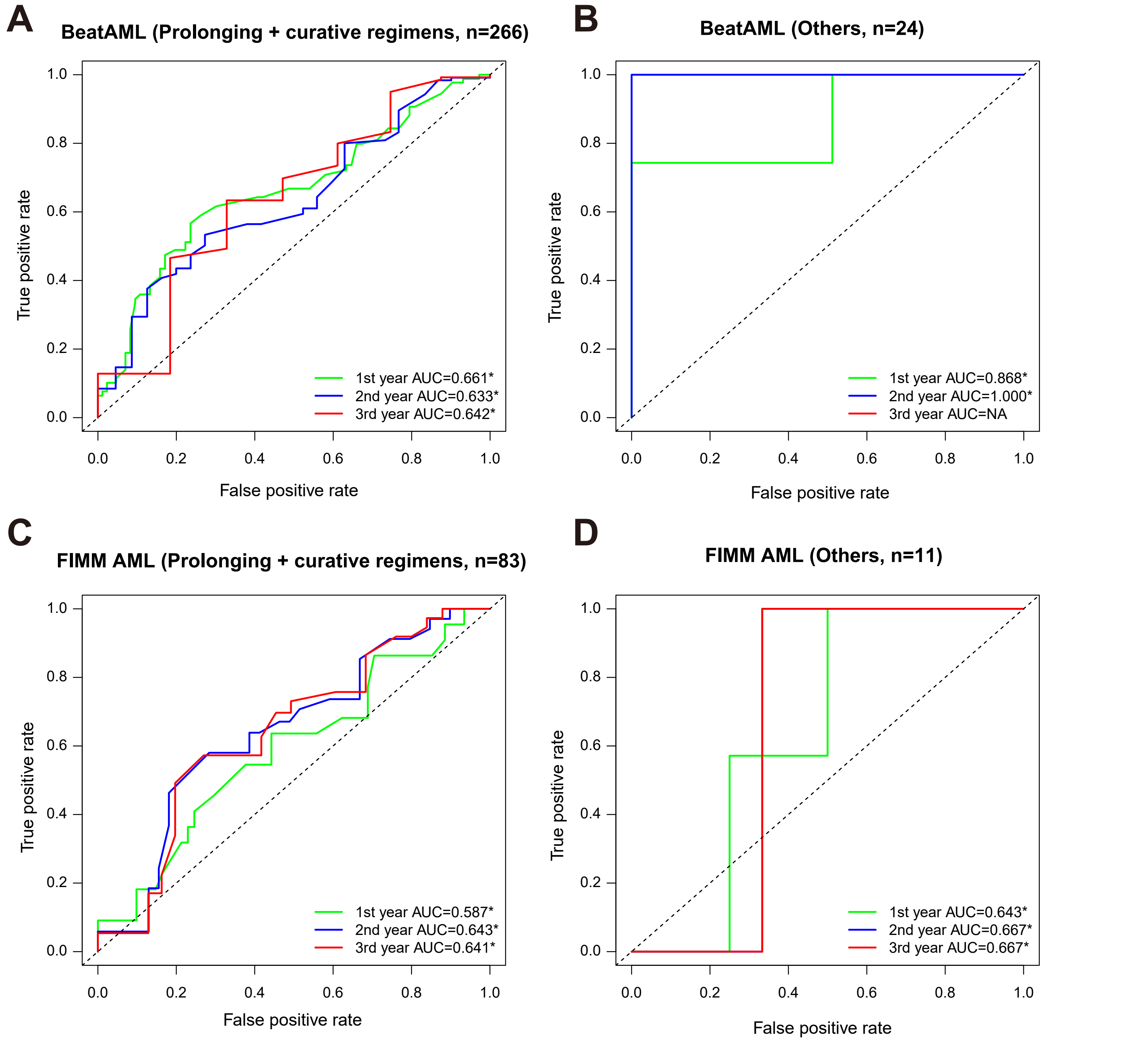

Supplementary Figure 10. The ROC curves of patients in the IPRP high and low-risk groups receiving different treatment modalities. (A) The prolonging and curative treatment in the BeatAML cohort. (B) Other treatment modalities (palliative care or unknown type) in the BeatAML cohort. (C) The prolonging and curative treatment in the FIMM AML cohort. (D) Other treatment modalities (palliative care or unknown type) in the FIMM AML cohort. The black diagonal line corresponds to the random classifier (ROC-AUC=0.5). * $p<0.05$ (permutation test). “NA” means there is not enough samples to perform prediction.

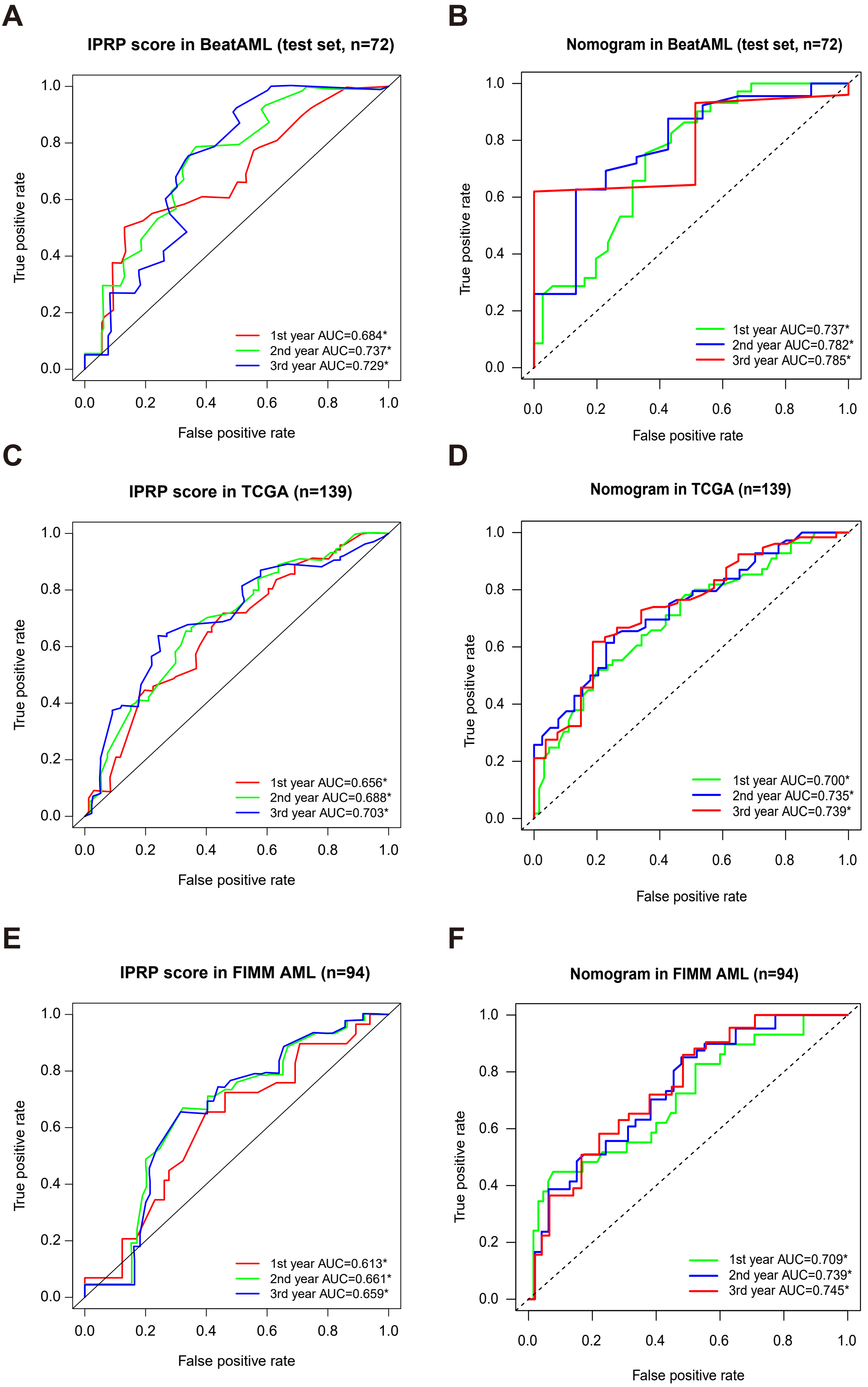

Supplementary Figure 11. The comparison of prognostic accuracy between IPRP score (left panels) and 4-factor nomogram including the IPRP score (right panels) in 3 test cohorts. The colored curves correspond to different follow-up time points in the survival data with ROC-AUC values. The black diagonal line corresponds to the random classifier (ROC-AUC=0.5). * p<0.05 (permutation test).

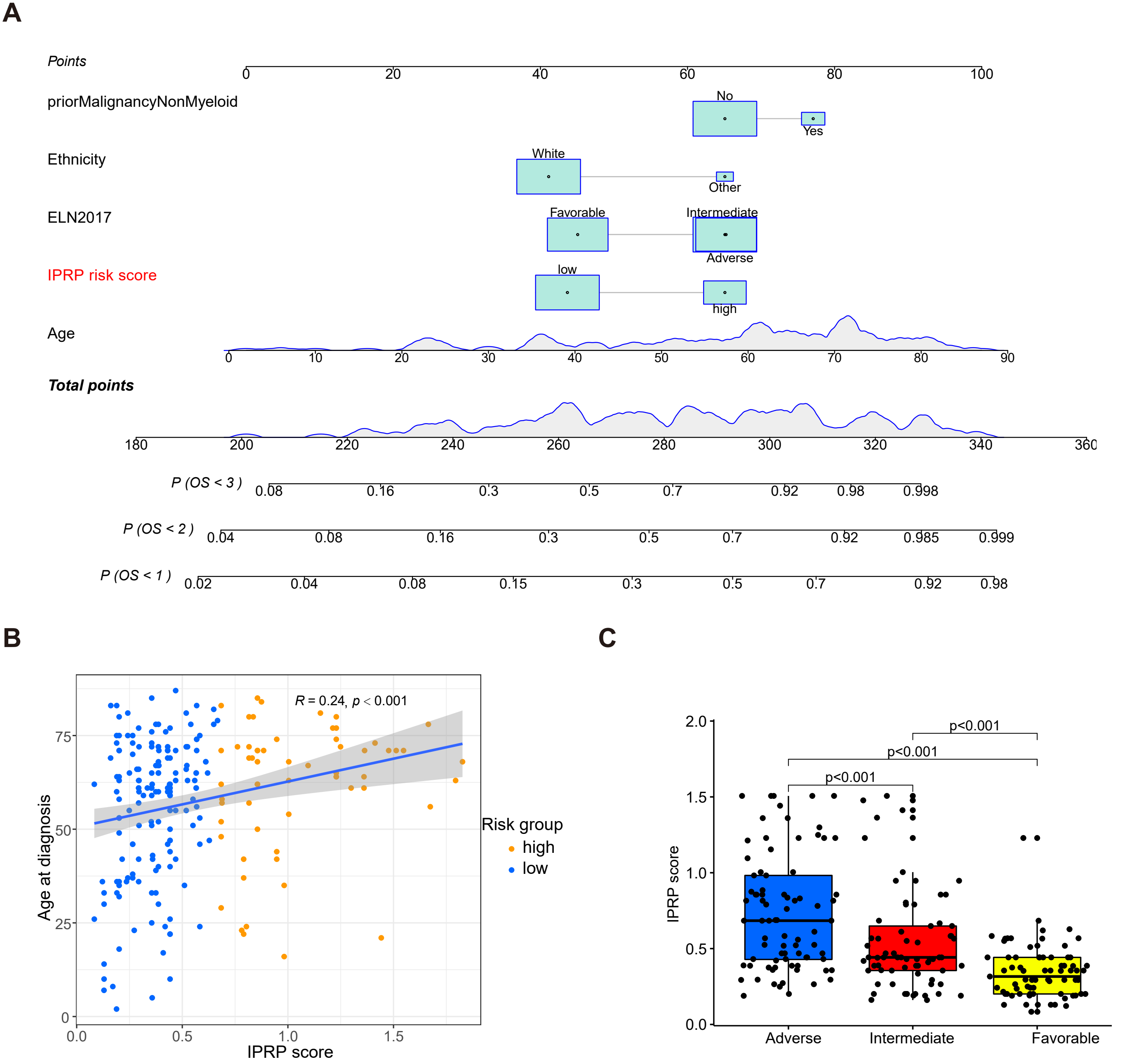

Supplementary Figure 12. (A) The 5-factor nomogram including the ELN2017 classification. (B) The association between the age at diagnosis and IPRP scores in the BeatAML cohort using the Spearman correlation coefficient. (C) The association between the ELN2017 and IPRP scores in the BeatAML cohort using the ANOVA test.

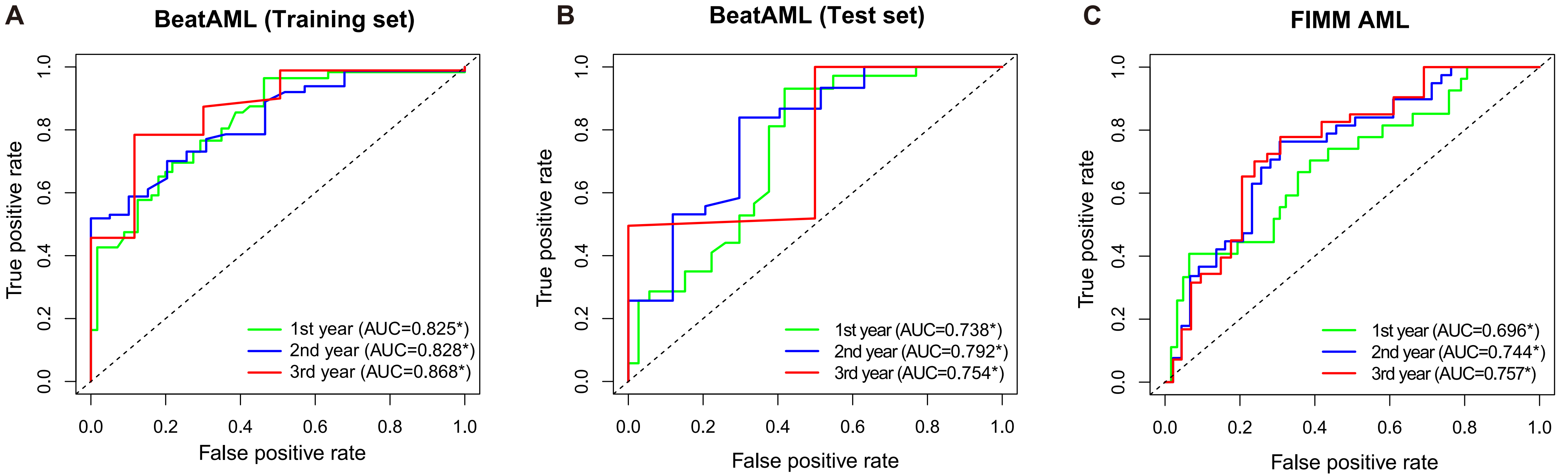

Supplementary Figure 13. ROC curves of the 5-factor nomogram that includes ELN2017 as an additional factor in the BeatAML (A) training and (B) test set; (C) FIMM AML cohort. * p<0.05 (permutation test).

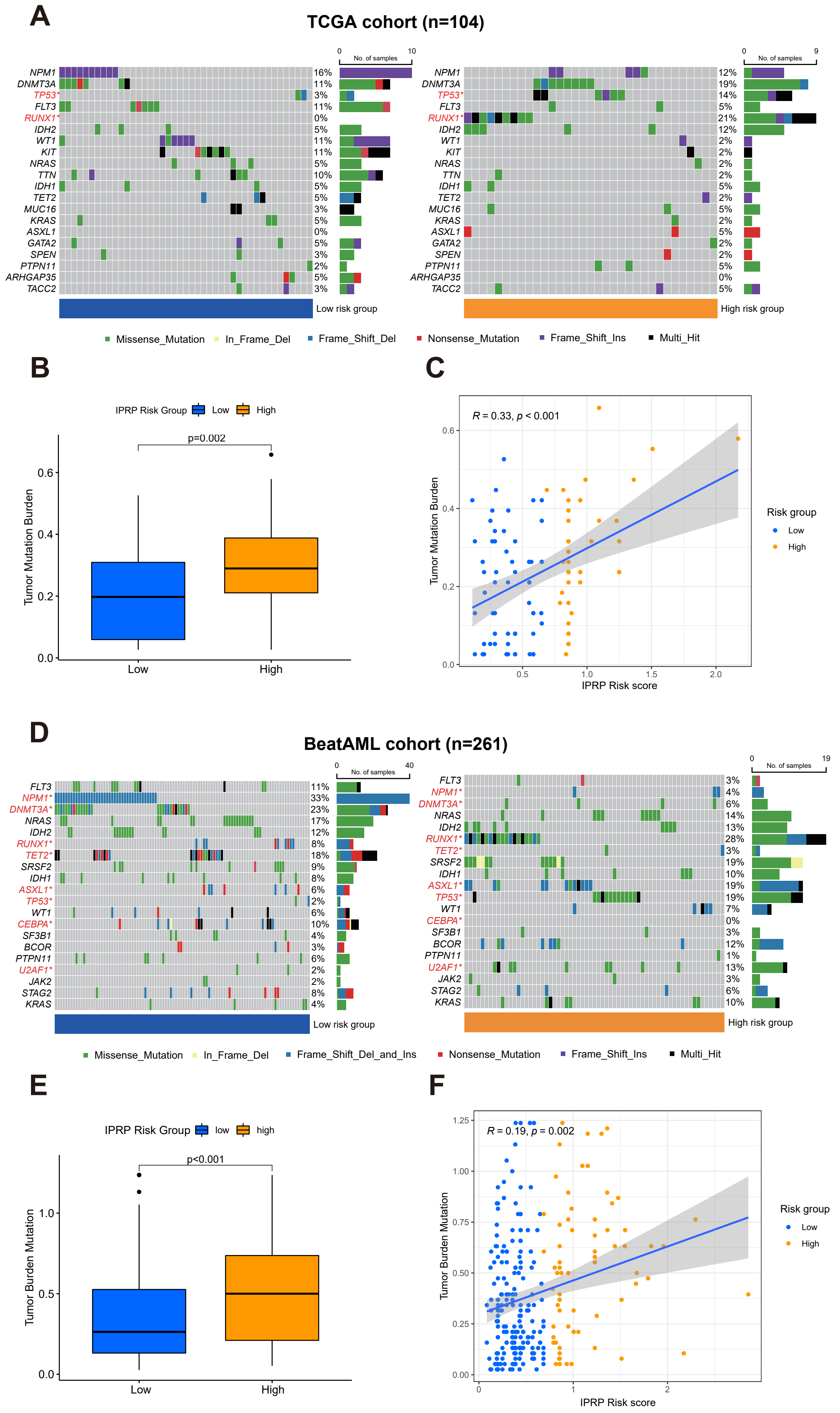

Supplementary Figure 14. Mutation analysis in the TCGA-AML and BeatAML cohorts. (A) The most frequent cancer gene mutations in the IPRP low and high risk groups in TCGA-AML cohort. The Chi-square test was used to compare the frequency of mutations between the two groups of AML patients. (B) TMB differences between the low and high risk groups in TCGA-AML cohort. Statistical significance was assessed with the Wilcoxon test. (C) Correlation analysis between IPRP score and TMB levels in TCGA-AML cohort. Statistical association was assessed with the Spearman correlation coefficient (R). (D) The most frequent cancer gene mutations in the IPRP low and high risk groups in BeatAML cohort. The Chi-square test was used to compare the frequency of mutations between the two groups of AML patients. (E) TMB differences between the low and high risk groups in BeatAML cohort. Statistical significance was assessed with the Wilcoxon test. (F) Correlation analysis between IPRP score and TMB levels in BeatAML cohort. Statistical association was assessed with the Spearman correlation coefficient (R).

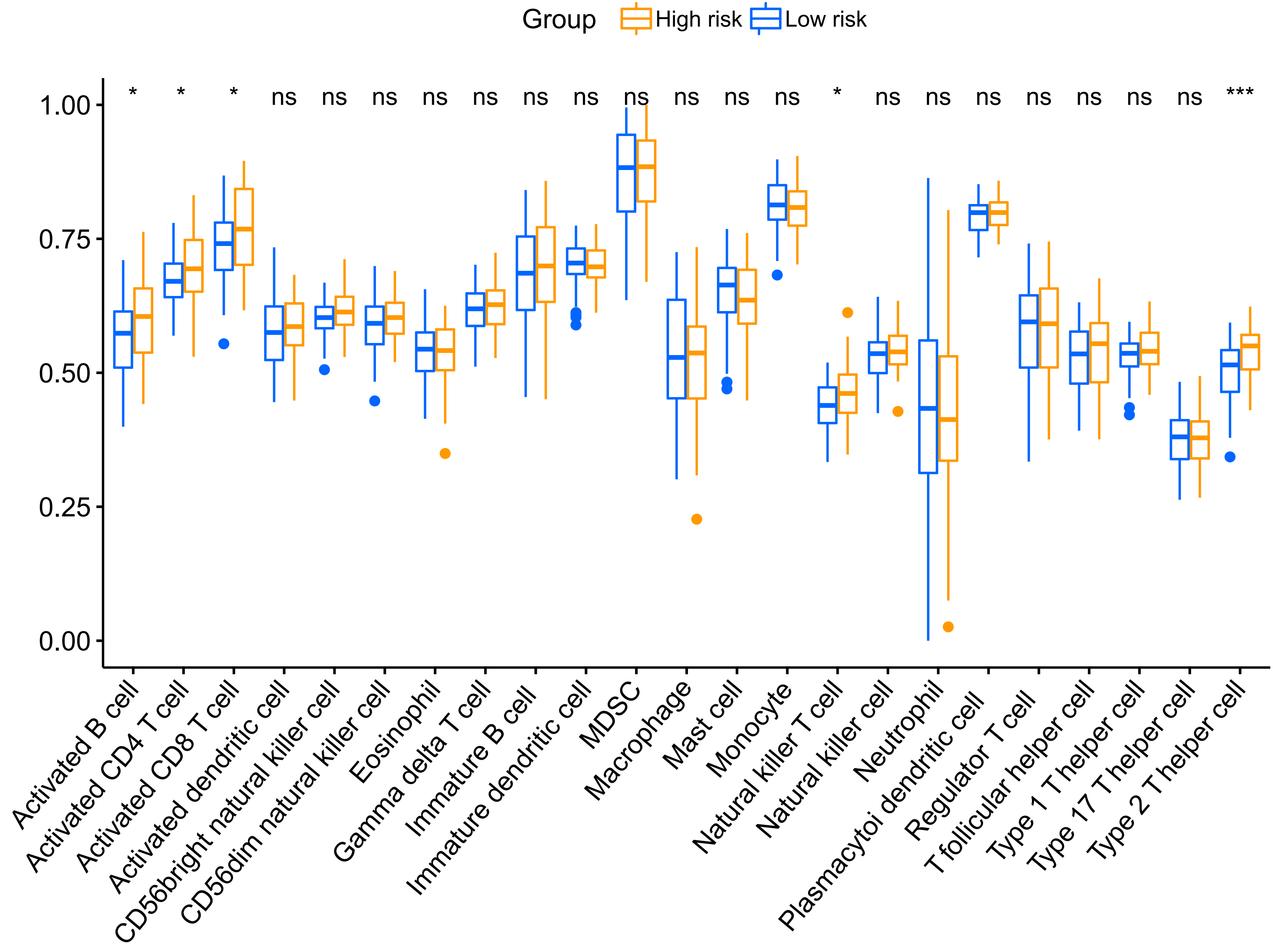

Supplementary Figure 15. Immune infiltration analysis of immune cells in TCGA-AML patients using ssGSEA. Benjamini and Hochberg method was used for adjusting for multiple testing. * adjusted p<0.05.

Cancer	adj.p-value	Hazard ratio
ACC	0.527	1.219(0.660−2.250)
BLCA	0.396	0.929(0.783−1.101)
BRCA	0.664	1.030(0.901−1.178)
CESC	0.369	0.815(0.522−1.273)
CHOL	0.377	0.813(0.514−1.286)
COAD	0.274	1.132(0.907−1.412)
ESCA	0.235	0.851(0.653−1.110)
GBM	0.758	0.941(0.637−1.388)
HNSC	0.131	0.821(0.635−1.061)
KICH	0.564	1.235(0.603−2.529)
KIRC	0.363	1.123(0.874−1.443)
KIRP	0.952	0.981(0.527−1.827)
LGG	<0.001	0.385(0.290−0.509)
LIHC	0.995	0.999(0.806−1.239)
LUAD	0.061	0.873(0.757−1.006)
LUSC	0.557	1.041(0.910−1.191)
MESO	0.037	0.840(0.713−0.989)
OV	0.193	1.115(0.946−1.314)
PAAD	0.400	0.940(0.815−1.085)
PCPG	0.457	0.497(0.078−3.143)
PRAD	0.614	1.266(0.506−3.165)
READ	0.749	1.091(0.642−1.854)
SARC	0.477	1.051(0.916−1.205)
SKCM	0.308	1.185(0.855−1.640)
STAD	0.861	0.982(0.800−1.205)
TGCT	0.898	1.141(0.154−8.436)
THCA	0.031	2.118(1.070−4.193)
UCEC	0.793	0.965(0.736−1.263)
UCS	0.742	1.089(0.655−1.811)
UVM	0.063	0.463(0.206−1.044)

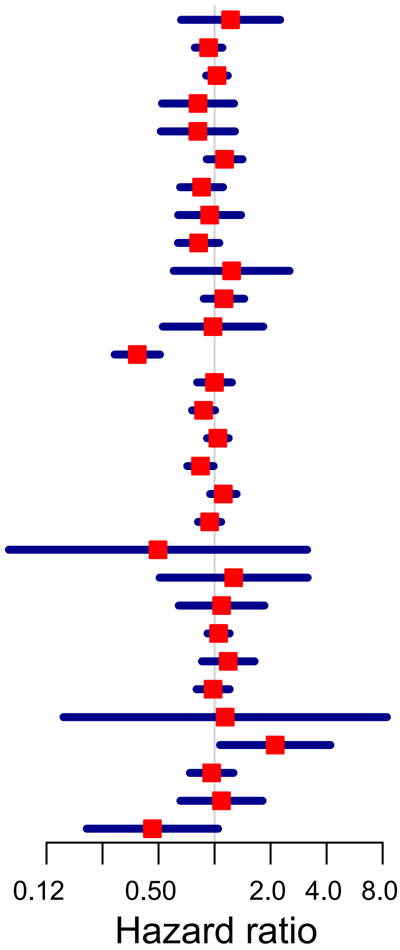

Supplementary Figure 16. Cox regression analysis of the 6 established immune subtypes in the TCGA-AML datasets. Benjamini and Hochberg method was used for adjusting for multiple testing. Red color indicates positive association and blue negative association.

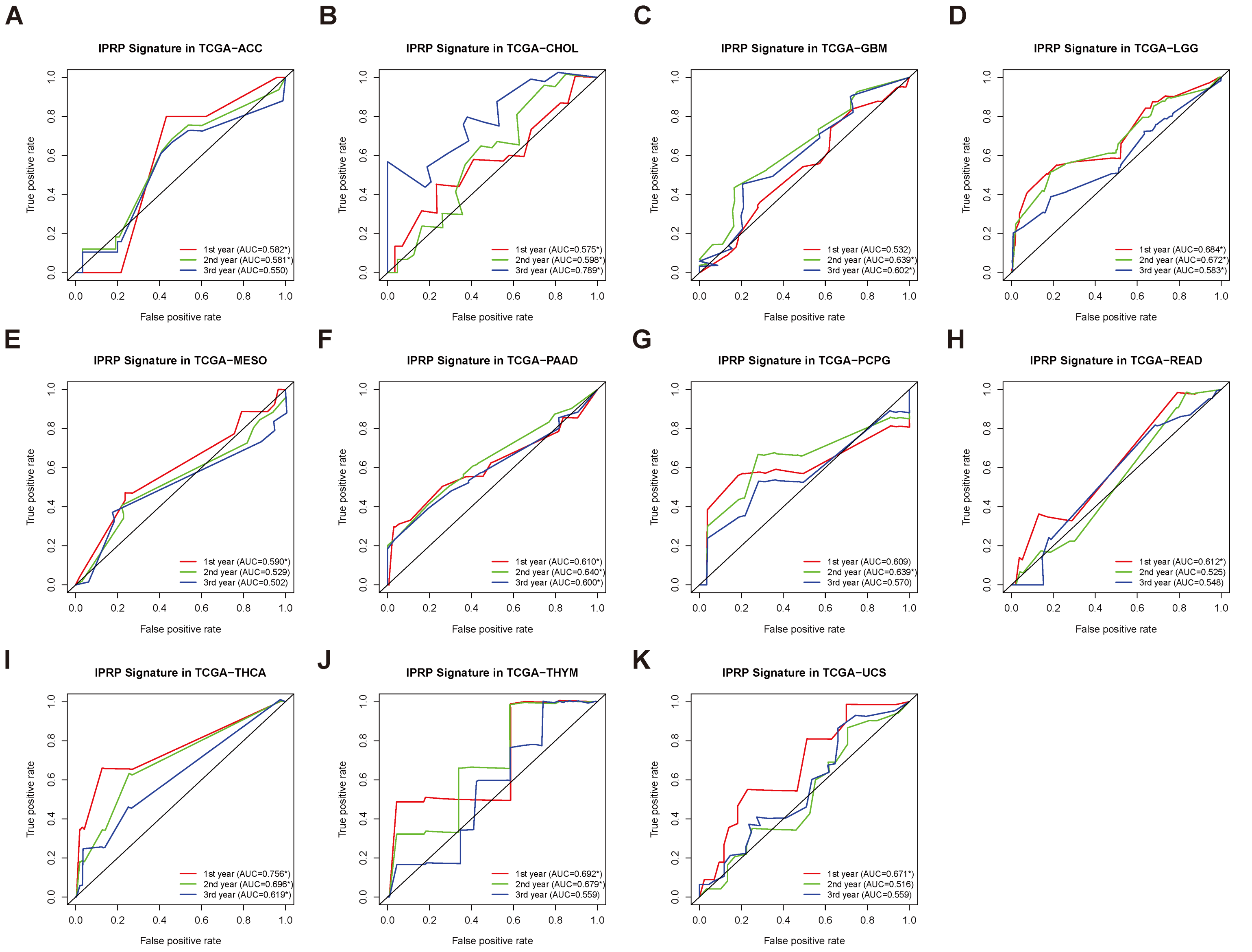

Supplementary Figure 17. TCGA cancer types with significant ROC-AUC values based on the IPRP score. * p<0.05, permutation test.

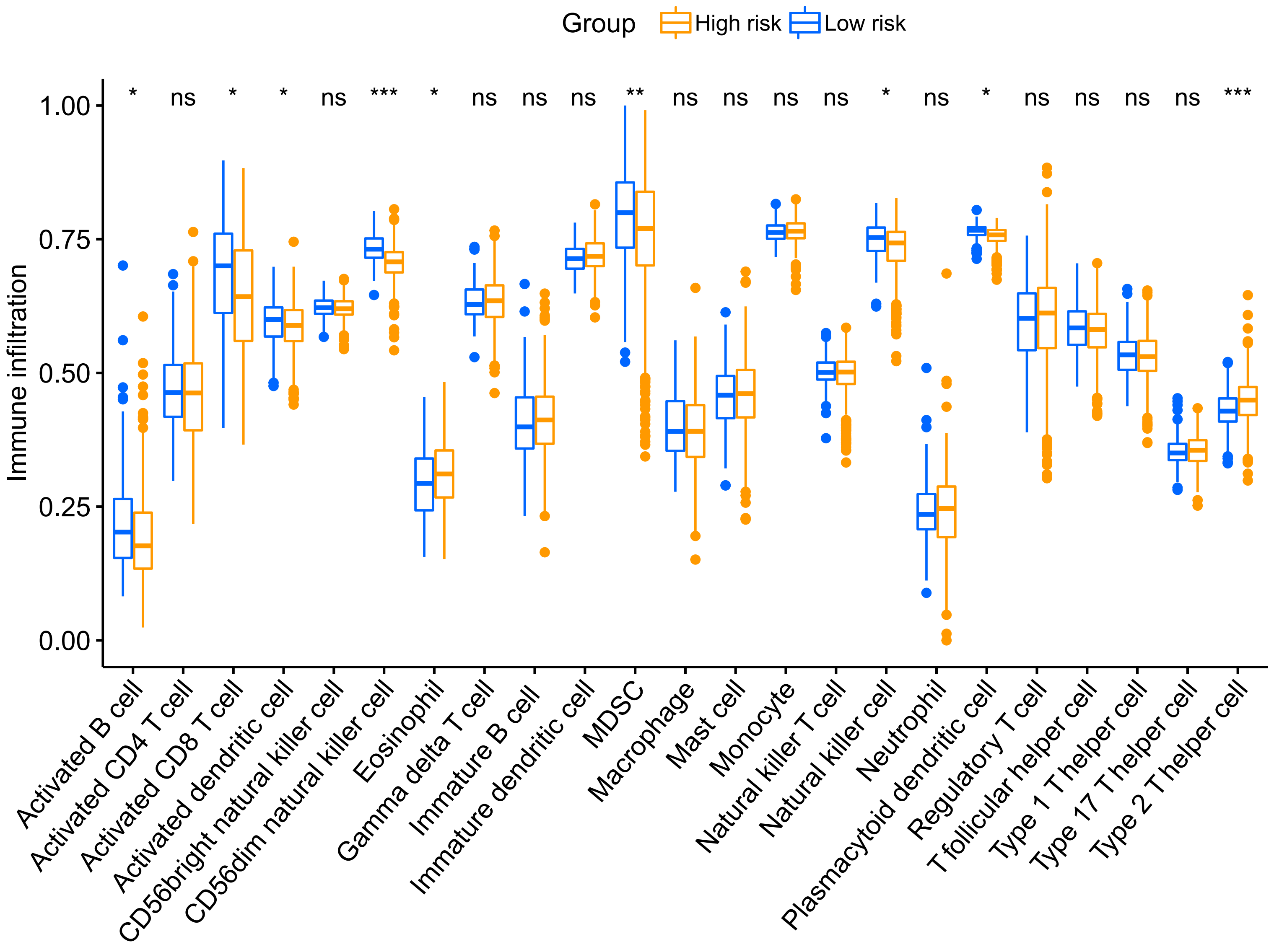

Supplementary Figure 18. Immune infiltration analysis of immune cells in TCGA-KIRC patients using ssGSEA. Benjamini and Hochberg method was used for adjusting for multiple testing. * adjusted p<0.05.

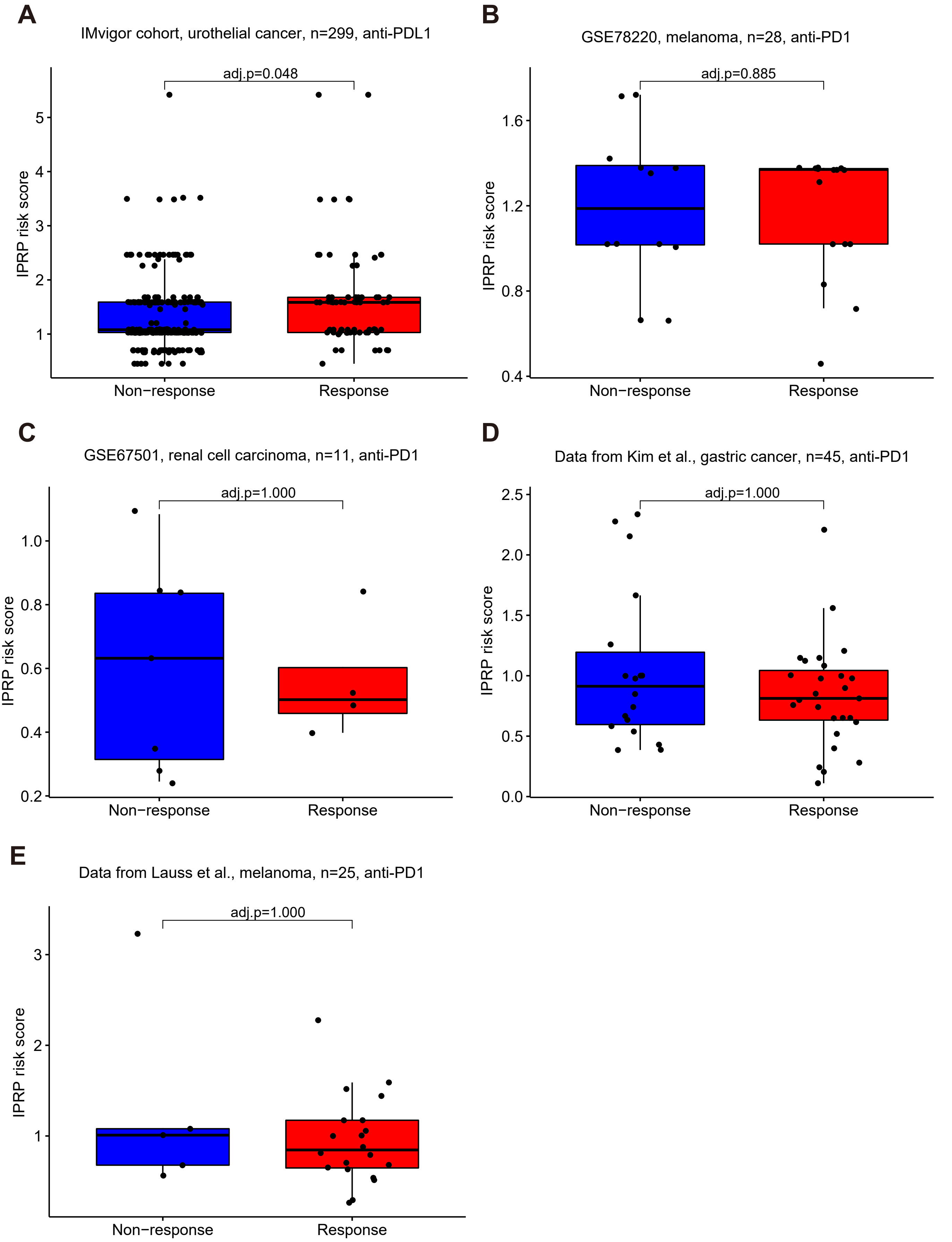

Supplementary Figure 19. Differences in the IPRP risk score between immunotherapy response groups in published datasets. Response differences were assessed with the Wilcoxon test, and p-values adjusted with Benjamini and Hochberg method.

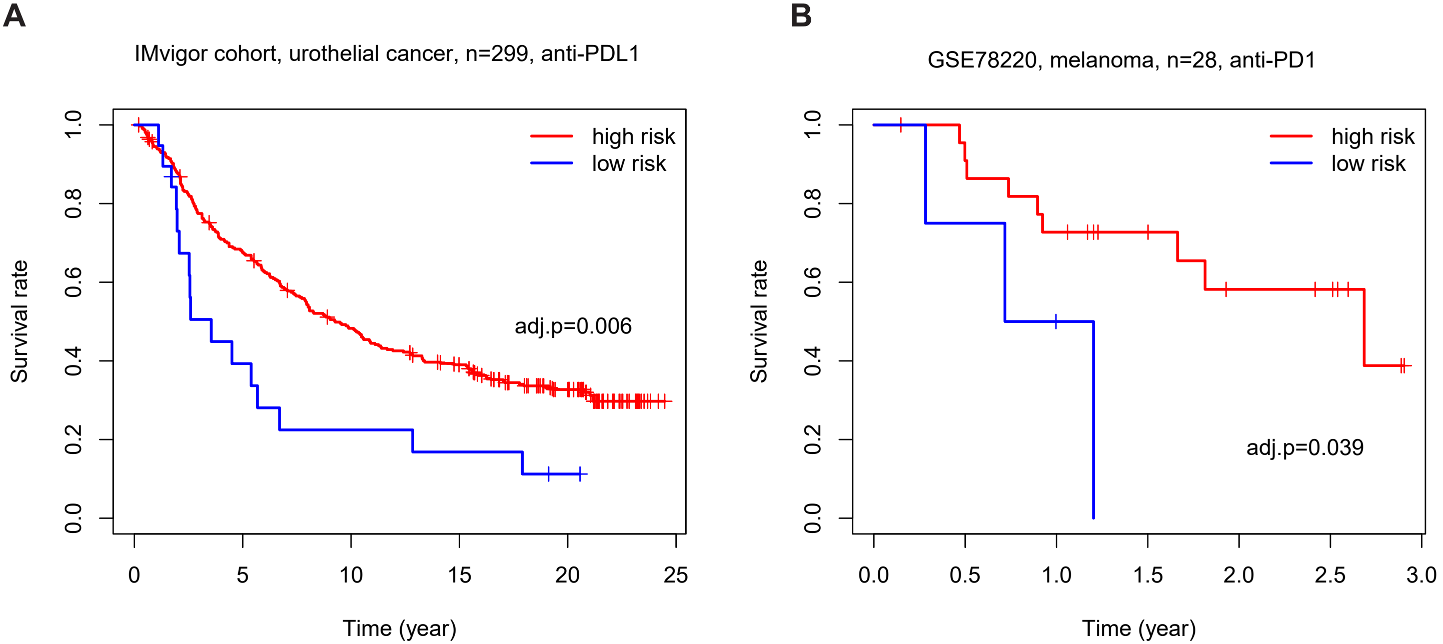

Supplementary Figure 20. Survival differences in the IMvigor and GSE78220 cohorts between the risks groups defined based on IPRP score. Survival differences were assessed with Log rank test, and p-values adjusted with Benjamini and Hochberg method.
